# Chemo‐proteomics in antimalarial target identification and engagement

**DOI:** 10.1002/med.21975

**Published:** 2023-05-26

**Authors:** Brodie L. Bailey, William Nguyen, Alan F. Cowman, Brad E. Sleebs

**Affiliations:** ^1^ The Walter and Eliza Hall Institute of Medical Research Melbourne Victoria Australia; ^2^ Department of Medical Biology The University of Melbourne Melbourne Victoria Australia

**Keywords:** antimalarial, chemical probe, malaria, target engagement, target identification

## Abstract

Humans have lived in tenuous battle with malaria over millennia. Today, while much of the world is free of the disease, areas of South America, Asia, and Africa still wage this war with substantial impacts on their social and economic development. The threat of widespread resistance to all currently available antimalarial therapies continues to raise concern. Therefore, it is imperative that novel antimalarial chemotypes be developed to populate the pipeline going forward. Phenotypic screening has been responsible for the majority of the new chemotypes emerging in the past few decades. However, this can result in limited information on the molecular target of these compounds which may serve as an unknown variable complicating their progression into clinical development. Target identification and validation is a process that incorporates techniques from a range of different disciplines. Chemical biology and more specifically chemo‐proteomics have been heavily utilized for this purpose. This review provides an in‐depth summary of the application of chemo‐proteomics in antimalarial development. Here we focus particularly on the methodology, practicalities, merits, and limitations of designing these experiments. Together this provides learnings on the future use of chemo‐proteomics in antimalarial development.

**Abbreviations**
ABPP: activity‐based protein profiling
ACT: artemisinin combination therapy
AfBPP: affinity‐based protein profiling
ALDH1: aldehyde dehydrogenase family 1
ALLN: N‐acetyl‐Leu‐Leu‐Norleu‐al
ART: artemisinin
ATC: aspartate transcarbamoylase
AzT: TAMRA azide
AzTB: TAMRA biotin azide
CDK2: cyclin dependent kinase
CEPT: choline/ethanolamine phosphotransferase
CETSA: cellular thermal shift assay
CK1: casein kinase
CnBr: cyanate ester
CQ: chloroquine
crapOME: contaminant repository for affinity purification
CSP: circumsporozoite protein
CuAAC: copper‐catalyzed azide‐alkyne cycloaddition
DARTS: drug‐affinity responsive target stability
DFO: desferrioxamine
DFP: deferiprone
DHODH: dihydroorotate dehydrogenase
DV: digestive vacuole
emPAI: exponentially modified protein abundance index
HA: hemagglutinin A
HDP: hemoglobin derived products
HEA: hydroxyethyl amine
HKMT: histone lysine methyltransferases
HQ: hydroxychloroquine
ICAT: isotope‐coded affinity tagging
IEDDA: inverse‐electron demand Diels–Alder
ITDR: isothermal drug response
Itraq: isobaric tagging for relative and absolute quantification
IVE‐GWAS: in vitro evolution—genome wide association studies
LC/MS/MS: liquid chromatography‐tandem mass spectrometry
MFQ: mefloquine
MS: mass spectrometry
Myr‐CoA: Myristoyl‐Coenzyme A
NHS: N‐hydroxysuccinamide
NMT: N‐myristoyltransferase
PAL: photoaffinity labeling
PfATC: *Plasmodium falciparum* aspartate transcarbamoylase
PfCDPK1: *Plasmodium falciparum* calcium‐dependent protein kinase
PfDHFR‐TS: *Plasmodium falciparum* dihydrofolate reductase‐thymidylate synthase
PfENT4: *Plasmodium falciparum* Equilibrative Nucleoside Transporter
PfMDR1: *Plasmodium falciparum* multidrug resistance protein
PfOAT: *Plasmodium falciparum* ornithine aminotransferase
PfPNP: *Plasmodium falciparum* purine nucleoside phosphorylase
PfPyKII: *Plasmodium falciparum* pyruvate kinase II
PfSPP: *Plasmodium falciparum* Signal Peptide Peptidase
PI4K: phosphatidylinositol 4‐kinase
PKG: cGMP‐dependent kinase
PM: plasmepsin
PMIX: plasmepsin IX
PMX: plasmepsin X
PQ: primaquine
PROTACs: proteolysis‐targeting chimeras
QR2: quinine oxidoreductase 2
RBC: red blood cell
RING: really interesting new gene
Sal A: Salinipostin A
SAR: structure–activity relationship
SILAC: stable isotope labeling with amino acids in culture
SPAAC: strain‐promoted azide‐alkyne cycloaddition
SPR: surface plasmon resonance
SPROX: stability of proteins from rates of oxidation
TER: tetraethylrhodamine
TMT: tandem mass tagging

## 1 INTRODUCTION

Malaria is a parasitic protozoan disease that causes a huge burden on human well‐being worldwide.^1^ Over 200 million people are infected with the disease annually, resulting in approximately 619,000 deaths in 2021.^2^ Restricted primarily to tropical regions, malaria is caused by the transmission of *Plasmodium* parasites by the bite of the female *Anopheles* mosquito. While a number of *Plasmodium* species have the potential to cause human disease, *P. falciparum* and *P. vivax* have the most significant impact on mortality and morbidity.^2^ The management of malaria consists of mosquito intervention methods and antimalarial chemotherapy which collectively, have resulted in a significant reduction in morbidity and mortality over the past 20 years.^3^ Unfortunately, resistance to most currently available antimalarials has been observed in *P. falciparum*, including the front‐line Artemisinin Combination Therapies (ACTs).^4^ As such, some drug classes used to treat the disease are no longer recommended for clinical use.^5^, ^6^ To curb the onset of resistance, world health authorities have prescribed that new antimalarials have a novel chemotype and target a mechanism of action not previously reported.^7^ More recently, a pioneering RTS,S/AS01 vaccine based on the circumsporozoite protein (CSP) has been approved for use in children in areas of high transmission.^2^ However, the efficacy of the vaccine is just 36% over 4 years of monitoring.^8^ Therefore, antimalarial chemotherapies will remain at the forefront of disease treatment and control.

To discover new antimalarial chemotypes, there has been an explosion in mass phenotypic high throughput screening of large compound libraries in the past 20 years.^9^, ^10^, ^11^ These screens have been primarily performed on the asexual erythrocytic stage of *P. falciparum* as this form of the parasite is the most tractable in the laboratory,^12^ although more recently, assays and platforms become available to screen both the sexual (both gametocyte and gamete)^13^ and liver sporozoite and schizont stages of the *P. falciparum* lifecycle.^14^, ^15^ Additionally, methods have been established to screen against the latent *P. vivax* hypnozoite.^16^, ^17^ The mass phenotypic screening effort has resulted in the identification of starting points that have led to the development of several clinical candidates, such as cipargamin (KAE609), ganaplacide (KAF156), and MMV048, undergoing Phase II trials.^18^, ^19^, ^20^, ^21^ While phenotypic‐based screening has become the mainstay for the identification of new antimalarial chemotypes, target‐based screening has also uncovered starting points against genetically validated targets, for example, the dihydroorotate dehydrogenase (DHODH) inhibitor and Phase II clinical candidate DSM265.^22^, ^23^

Both phenotypic and target‐based drug discovery methods present their own unique challenges. For phenotypic drug discovery, once a hit molecule is identified, a key development task is to deconvolute the mechanism of action.^24^ While antimalarials can be developed without a fully described mechanism of action,^25^ establishing the target is highly desirable for the following reasons.^26^ First, the target can help define the target product profile by understanding the target pharmacology, and secondly, visualizing the compound in complex with a protein target can expedite the development via structural‐based design. For target‐based drug discovery, target engagement within the parasite is important to demonstrate the compound is indeed killing the parasite via the target. Target engagement is also crucial in validating the target of the phenotypic hit once it has been uncovered by target identification methods. Nevertheless, for either target or phenotypic approaches, the process of target identification and engagement is a vital aspect of antimalarial research and development.

The most extensively used approaches toward antimalarial target identification involve omic methods, which have been comprehensively reviewed elsewhere.^27^, ^28^, ^29^, ^30^, ^31^, ^32^ Briefly, these include genomic, metabolomic, and proteomic methods. Genomic methods in target deconvolution involve in vitro evolution of resistance to the compound of interest followed by genome‐wide association studies (IVE‐GWAS) or nucleotide expression profiling with microarray followed by compound treatment.^33^ IVE‐GWAS relies on culturing resistance to the compound of interest, which may not be possible if the compound elicits its pharmacological response via inhibition of multiple protein targets, pathways that are not genome‐encoded or host‐derived proteins. Metabolomics is another popular target deconvolution method whereby alterations to the parasite metabolome are detected following drug treatment, identifying pathways that are indirectly inhibited.^34^, ^35^ Generally, this method provides a top‐down analysis and requires further studies to elucidate the protein target(s). Global transcriptomics and proteomic methods follow a similar rationale with protein and mRNA levels monitored following drug treatment.^36^, ^37^

Chemo‐proteomic methods have recently emerged as a useful alternative to directly and unbiasedly detect the protein target(s) of antimalarial compounds discovered from phenotypic screening. Chemo‐proteomic techniques are an example of direct target identification methods in which the effects of compound–target interactions are measured directly, not through downstream events. Examples of such techniques include affinity binding techniques using pulldown probes and thermal stability profiling. Additionally, many of the unbiased chemo‐proteomic methods are adapted to biased methods to demonstrate compound engagement with a parasite protein target to assist with on‐target validation of the antimalarial under development.

This perspective will focus on the application of chemical biology methods in antimalarial target identification and target engagement. This appraisal of the field distinguishes itself from recent overviews^38^, ^39^, ^40^ by providing a detailed description of key examples using chemical biology techniques in antimalarial target identification and target engagement while discussing the advantages and limitations. This review aims to act as a guide for the development and application of chemical biology techniques in antimalarial target deconvolution and more broadly in antimalarial drug development.

## 2 PARASITE AND HOST‐SPECIFIC CONSIDERATIONS IN CHEMO‐PROTEOMICS

### 2.1 A complex lifecycle

A unique aspect of *Plasmodium* biology is its complex, multi‐host lifecycle. Human infection begins with the injection of infective sporozoites from *Anopheles* mosquitoes and these parasites make their way to the liver and invade hepatocytes, where they undergo schizogony or form dormant hypnozoites in the case of *P. vivax* and *P. ovale*.^41^ Liver schizonts release large numbers of merozoites into the bloodstream which invade red blood cells and begin the asexual blood cycle. A portion of these erythrocytic forms diverges into the sexual blood stage, forming gametocytes that can be consumed by the mosquito in a blood meal and develop in this definitive host which completes the cycle. Dramatic changes in parasite morphology and size occur across the different stages, and indeed within these stages. Consequently, the parasite proteome differs widely, as do potential drug targets.^42^ The importance of developing drugs that target all of these stages has been clearly underlined therefore efficient phenotypic screening methods and henceforth target identification is essential for drug development.^43^ Comparatively, proteomic sample preparation of *Plasmodium* is arduous and expensive in large quantities.^44^ Therefore, a formidable challenge for *Plasmodium* proteomic research has been the development of robust culturing conditions at significant scale for quality data.^44^ In the following Sections 2.1.1, 2.1.2, 2.1.3, 2.1.4, 2.1.5, parasite stage and host‐specific considerations are outlined for application in chemo‐proteomic methods.

#### 2.1.1 Liver stage

One of the most difficult malaria lifecycle stages to study is the liver stage as the cells are not easily maintained for long periods and at scales sufficient for chemoproteomic analysis. To begin, infective sporozoites must be isolated and purified from the salivary glands of female *Anopheles*' mosquitoes, requiring specialized insectary facilities.^45^ Additionally, the numbers of liver cells invaded by sporozoites is small and some species exhibit cell‐specific invasion. The rodent species *P. berghei* has been widely used to study the liver stages due to its ability to be cultured in human lung,^46^ human hepatoma,^47^ HeLa,^48^ and mouse hepatocyte cell lines.^49^ Culture of human infective species such as *P. falciparum*, *P. vivax*, and *P. ovale* has been achieved in primary liver hepatocytes, however, these host cells cannot be kept in continuous culture.^50^, ^51^, ^52^ The human hepatoma cancer cell HepG2‐A16 was subsequently used as a method to culture liver stage *P. vivax*, but cannot support the development of *P. falciparum*.^53^, ^54^ More recently, the HC‐04 hepatocyte line has been developed to culture both *P. falciparum* and *P. vivax* liver stages.^55^, ^56^ To study the dormant liver stages produced by *P. vivax* and *P. ovale*, the specialized hepatocyte line imHC is used as HC‐04 hepatocytes proliferate unrestrictedly and detach from the culture dish, limiting their use for long‐term hypnozoites.^57^ The lack of chemo‐proteomic studies on liver stage parasites reflects experimental challenges, for example, difficult culturing conditions and target deconvolution in the presence of abundant host cell proteins. More sensitive methods, therefore, need to be developed to study target identification/engagement in this stage. However, chemo‐proteomic experiments looking at parasite effector proteins in the host hepatocyte may be possible.

#### 2.1.2 Asexual blood stage

The *P. falciparum* and *P. knowlesi* erythrocytic stages are the most easily maintained stage *in vitro* with the development of robust culturing conditions that enable continuous culture.^58^, ^59^ However, standard static cultures cannot be routinely kept above 10% parasitemia, therefore, considerable scale is required for large proteomic experiments.^60^ In contrast, no continuous culturing conditions exist for *P. vivax* parasites and samples must be derived directly from human infections, further complicating the species’ chemo‐proteomic study.^61^ Consequently, the majority of the chemo‐proteomic research has been performed on the *P. falciparum* asexual blood stages.

Continuous in vitro cultures of *Plasmodium* are characteristically asynchronous in their lifecycle.^58^ Protein expression can be highly stage‐specific and is fundamentally linked with stage‐specific activity observed in most antimalarial compounds. Once the stage of arrest is established, the specificity of proteomic data is enhanced with samples generated from synchronized parasites obtained through a range of methods. Sorbitol ring synchronization leads to purified ring stage cultures via stage‐specific permeability pathways.^62^ Mid‐trophozoite, schizonts, and gametocytes, on the other hand, can be purified using a magnetic resin that attracts the iron‐containing hemozoin complexes resulting from hemoglobin digestion in the parasite.^63^ Finally, centrifugation‐based purifications such as Percoll gradients can also be used to separate these stages according to their relative density.^64^

#### 2.1.3 Transmission stages

Gametocyte culturing conditions are analogous to the asexual erythrocytic stage and therefore can also be suitable for proteomic research.^13^, ^65^, ^66^, ^67^, ^68^, ^69^ Until recently, several issues have made their production at scale more difficult. The small numbers of asexual parasites that commit to this pathway (~5%) and the progressive loss of a culture's ability to produce gametocytes have been a bottleneck to production.^70^, ^71^ However, a recently reported CRISPR/Cas9‐engineered gametocyte producer line enables high sexual commitment rates (75%) for larger scale production.^72^ The inducible overexpression of the sexual commitment factor GDV1 greatly improves the control and yield of sexual forms for use in transmission research.^72^ Methods to separate early (I–III) and late (IV and V) gametocytes have been established.^13^, ^69^ Therefore, as with the other stages of the lifecycle, phenotyping, and establishment of early versus late‐stage gametocyte activity should be aligned with an effective proteomic study.

In vitro methods to culture and purify the remainder of the transmission stages that occur in the mosquito have been developed but at small scales. Exflagellation, or the formation of gametes from gametocytes, can be achieved through parasite resuspension in fetal bovine serum at pH 8.^73^ In vitro and ex vivo culturing of ookinetes is most common in *P. berghei*.^74^, ^75^ This is because the efficiency of conversion of *P. falciparum* parasites to mature ookinetes in vitro is very low compared to in vivo.^76^, ^77^ Maturation from ookinete to oocyst requires a complex coculture system with *Drosophila* cells and Matrigel substrate.^78^ Overall, the chemo‐proteomic study of the mosquito stages, particularly in *P. falciparum*, suffers from challenges in obtaining sufficient material and at the correct stage for a sensitive quantitative study. Improvements in culturing conditions, workup, and instrument sensitivity will aid in future proteomic work.

#### 2.1.4 Sub‐proteomics

In some cases, phenotypic indications such as timing and stage of antimalarial activity can provide clues as to the mechanism of action. For example, antimalarials with a delayed death phenotype (activity >48 h) are known to target the development of apicoplast organelles in daughter parasites.^79^, ^80^, ^81^ If such a hypothesis is known, the resolution and specificity of chemoproteomic results can be improved by obtaining sub‐proteomic extracts from isolated organelles. This has been achieved for the analysis of the food vacuole,^82^ micronemes,^83^, ^84^ and nucleus^85^ with differential centrifugation followed by density gradient separation. There are also analogous methods to isolate the mitochondria and apicoplast through nitrogen cavitation followed by density gradient separation, but these have not yet been applied to proteomic research.^86^

#### 2.1.5 Human host erythrocytes

As an obligate parasite, a major challenge in *Plasmodial* proteomic research is the contamination of host proteins. The blood and liver stages are encased in their host cell as well as a parasitophorous vacuole membrane. High abundance erythrocyte proteins, in particular hemoglobin, can mask the low abundance of parasite proteins.^44^ To avoid this, erythrocytic parasites can be purified with saponin lysis which selectively disrupts the erythrocyte membrane while leaving both the parasite and parasitophorous membrane.^87^ However, this does not fully resolve these issues as the parasites themselves break down hemoglobin and store by‐products such as hemozoin and hemoglobin‐derived products (HDPs) which can also complicate the sensitivity of proteomic studies.^88^ Therefore, for the proteomic study of *Plasmodium* careful consideration of protein extraction conditions should be taken. For example, traditional lysis buffers containing urea, thiourea, and DTT are thought to disrupt the food vacuole and thus release HDP, while freeze‐thaw lysis does not.^88^ However, the removal of erythrocyte proteins may not always be desirable and proteomics can be performed on parasitized red blood cells to identify potential human target proteins. For example, it is predicted that around 280 proteins of parasite origin are exported to the host erythrocyte with roles in immune avoidance and host cell remodeling.^89^, ^90^, ^91^ 13%–23% of these exported proteins are known to be essential, although no drugs are known to target these proteins as of yet, these could potentially be targets of antimalarials and require target deconvolution studies.^91^

## 3 CHEMICAL PROBES

One of the most widely applied chemical biology reagents in antimalarial target identification is the chemical probe. For the purposes of target identification, a chemical probe is a reagent used to purify or pulldown target proteins from complex mixtures by means of affinity or activity‐based protein profiling (AfBPP or ABPP). AfBPP leverages the intrinsic affinity of a compound of interest, acting like a bait. ABPP uses a slightly revised principle, relying on a reactive warhead that targets specific residues in the target active site. Often this is used to assess enzymatic families that have conserved catalytic residues, such as serine hydrolases, cysteine proteases, aspartyl, and glutamyl glycosidases.^92^ Unlike AfBPP, the target protein becomes covalently linked to the reactive warhead of the ABPP chemical probe, and therefore is irreversibly modified.

**Figure 1:**
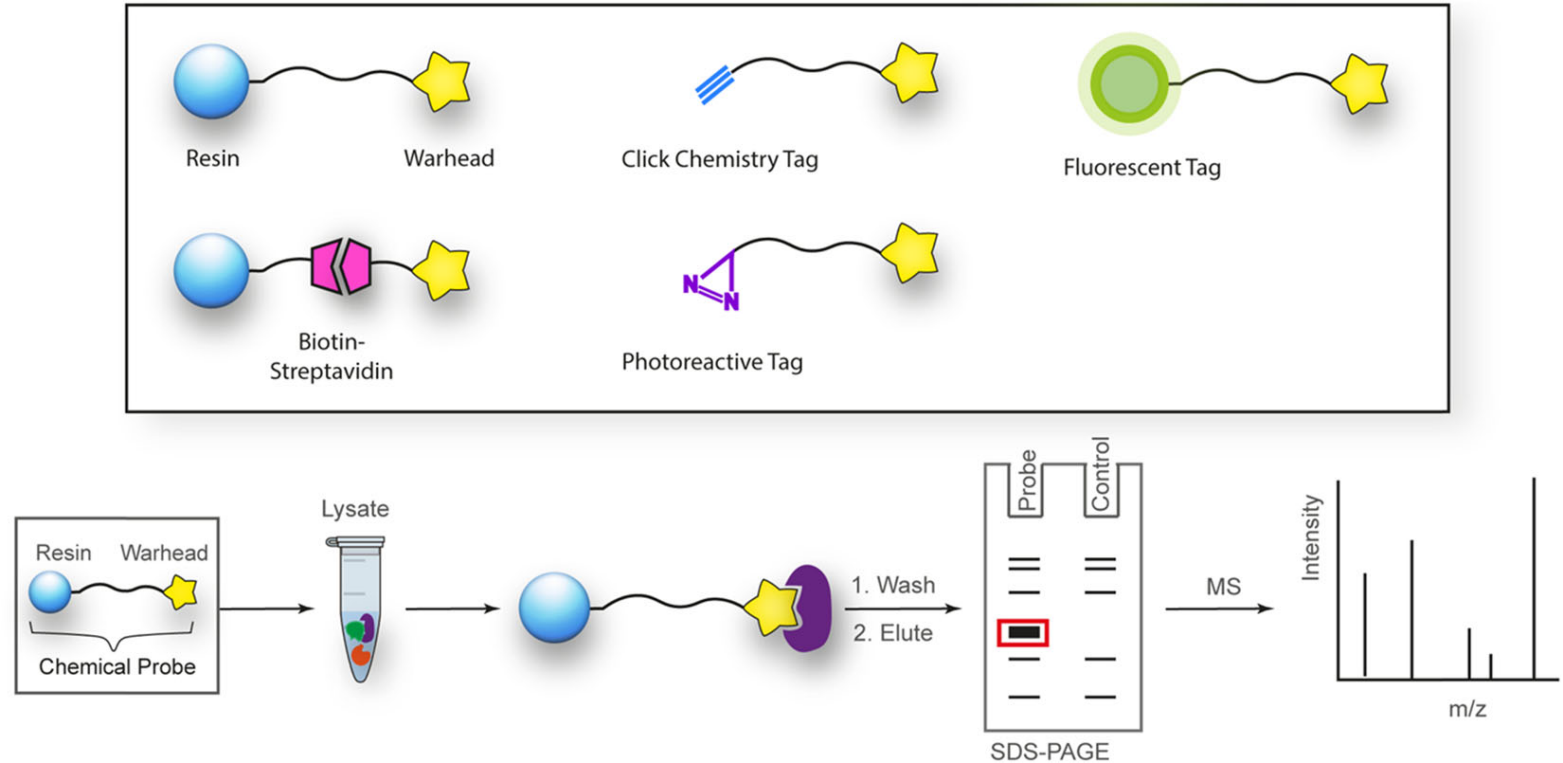
Structures and workflow of chemical probes used for target deconvolution. A range of chemical probe types can be employed for target elucidation, including resin immobilized probes, biotin‐streptavidin‐linked probes, fluorescent tag‐linked probes, and finally, probes with click chemistry and photoreactive tags. Chemical probes are constructed by linking the drug moiety to a solid support resin. The cellular lysate is applied to the resin to identify binding proteins. Rigorous washing steps reduce the levels of nonspecific, leaving only high‐affinity binders attached to the resin. The proteins are separated by SDS‐PAGE and are characterized either by western blot analysis or mass spectrometry. [Color figure can be viewed at wileyonlinelibrary.com]

The construction of a chemical probe is achieved by conjugating a target‐interacting warhead via a linker to a solid support or functional tag (Figure 1). The sophistication of the compound conjugation or labeling method has rapidly expanded from simple resin immobilization to employing click chemistry, photo‐crosslinking, and even bioorthogonal chemistry. These methods utilize a specialized set of specific chemical reactions there are highly efficient for the conjugation of small molecules. The positioning of the linker or functional label on the compound structure is key to maintaining the target protein binding affinity or activity.^93^ Typically, the correct positioning of the label requires prior knowledge of the structure–activity relationship (SAR) performed by rational design, or if the protein target is known, visualization of the compound in complex with the protein target may guide the appropriate location to append the label. Confirmation of the probe's activity is often desired; however, the addition of large linkers may preclude cellular permeability and therefore this measurement may not be useful. Instead, a lower molecular weight handle can be utilized as a surrogate to ascertain the activity of the probe.

Once the pulldown is complete, protein characterization methods differ depending on the level of knowledge of the target. Target validation in parasites typically utilizes a biased approach whereby an antibody to the target or an engineered parasite line expressing the labeled target is used for detection by Western blot. For unbiased approaches, these methods rely extensively on mass spectrometry and quantitative proteomics to identify targets (described in Section 5).^94^ Poly‐pharmacology is a common feature of phenotypically discovered drugs, and indeed the most effective antimalarials for combatting drug resistance.^95^ An unbiased approach can be used to identify such features, therefore, it is well suited for the identification of antimalarial targets.^95^ Similarly, chemical probes have the ability to identify off‐target binding which can have important implications for understanding human toxicity that may be observed.^96^ For example, an antimalarial chemical probe has led to a proposal for the toxicity observed with chloroquine (discussed later).^97^ Chemical probes are pharmacologically relevant, concentration‐dependent, and can be used with almost any cell type.

A limitation of AfBPPs and ABPPs is the propensity toward detecting false positives. For AfBPP, since many antimalarials (and indeed most other drug‐like compounds) have some degree of hydrophobicity, they are therefore predisposed to nonspecific protein interactions.^98^ Furthermore, AfBPPs and ABPPs are typically used at high concentrations that are not physiologically relevant increasing the likelihood of detecting false‐positive proteins. Distinguishing between nonspecific binding and true low‐affinity binders can be difficult, underpinning the importance of high‐affinity probes in addition to careful probe design and inclusion of appropriate vehicle and negative controls.^99^, ^100^ On the other hand, the reactive warhead on ABPP probes may lead to the modification of nontarget proteins. Due to these challenges, poorly characterized and nonselective probes have marred the reliability of research in this field.^101^ A need for emphasis on high‐quality chemical probes prompted the release of minimum standards for chemical probes by the Chemical Probes Portal.^102^ Here, it is recommended that probes should have well‐characterized in vitro activity, with a suitably structurally analogous inactive control, profiling of potential off‐target activity, and finally, evidence of cell permeability.^102^ Finally, while this method is widely applicable to cell types, it is largely limited to soluble proteins.^103^ While membrane proteins on rare occasions are suitable for both AfBPP and ABPP, they first require treatment of cells with an ionic nondenaturing detergent to release them from the surrounding membrane. Optimal solubilization conditions are difficult to predict without prior knowledge of the target or the parasite phenotype upon antimalarial treatment, as discussed in Section 2.

### 3.1 Affinity and activity based protein profiling

#### 3.1.1 Chemical probe immobilization techniques

##### Resin immobilization

Resin immobilized chemical probes are the simplest and most classical design. Resins are typically polymeric solid supports such as Sepharose (agarose) functionalized with a suitable reactive group, such as *N‐*hydroxysuccinamide (NHS) or cyanate ester (CNBr). The warhead is covalently attached via a linker to resin beads in an orientation that allows it access to the active site of target proteins.^104^ The workflow (Figure 1) generally involves the incubation of these chemical probes with cellular or tissue extracts, followed by extensive washing to remove nonspecific interactions. For AfBPP, remaining strong binders are eluted from the resin and separated by SDS‐PAGE at which point enriched protein bands can be identified compared to an inactive control probe. Elution conditions can include excess unlabeled drug to further assure the specificity of the binding proteins. ABPP results in irreversible protein binding therefore elution is not possible. Proteins are prepared for proteomics with on‐bead trypsin digestion, or alternatively, chemically, enzymatically, and photolytically cleavable linkers can be used to release the protein from its solid support.

##### Streptavidin immobilization

The activity of the probe may be impeded by the process of immobilization. To accommodate indirect affinity purification with such molecules, functional tags such as biotin can be used.^105^ A high‐affinity interaction between biotin and streptavidin (*K*_d_ ≈ 10^−14^ M) enables enrichment and immobilization when the latter is immobilized to an agarose resin.^106^, ^107^ In some cases,^108^ these biotin‐labeled probes are cell permeable and can be developed for use in live cells where the probe is captured following the cellular lysis.^98^

##### Bioorthogonal immobilization

A major advancement in the field of chemical probes is the development of robust bioorthogonal reactions; those that proceed within a cellular context without altering normal biochemistry.^109^ These reactions require complete chemoselectivity against a horde of cellular functional groups and must proceed rapidly at low temperatures in aqueous media.^110^ Coined by Sharpless et al.,^111^ “click chemistry” reactions have dominated this space. These reactions “follow nature's lead” and join modular units through highly specific and biocompatible chemical reactions.^111^ Common click chemistry reactions include the copper‐catalyzed azide‐alkyne cycloaddition (CuAAC), strain‐promoted azide‐alkyne cycloaddition (SPAAC), and the inverse‐electron demand Diels–Alder (IEDDA) using a strained alkene and tetrazine.^112^

**Figure 2:**
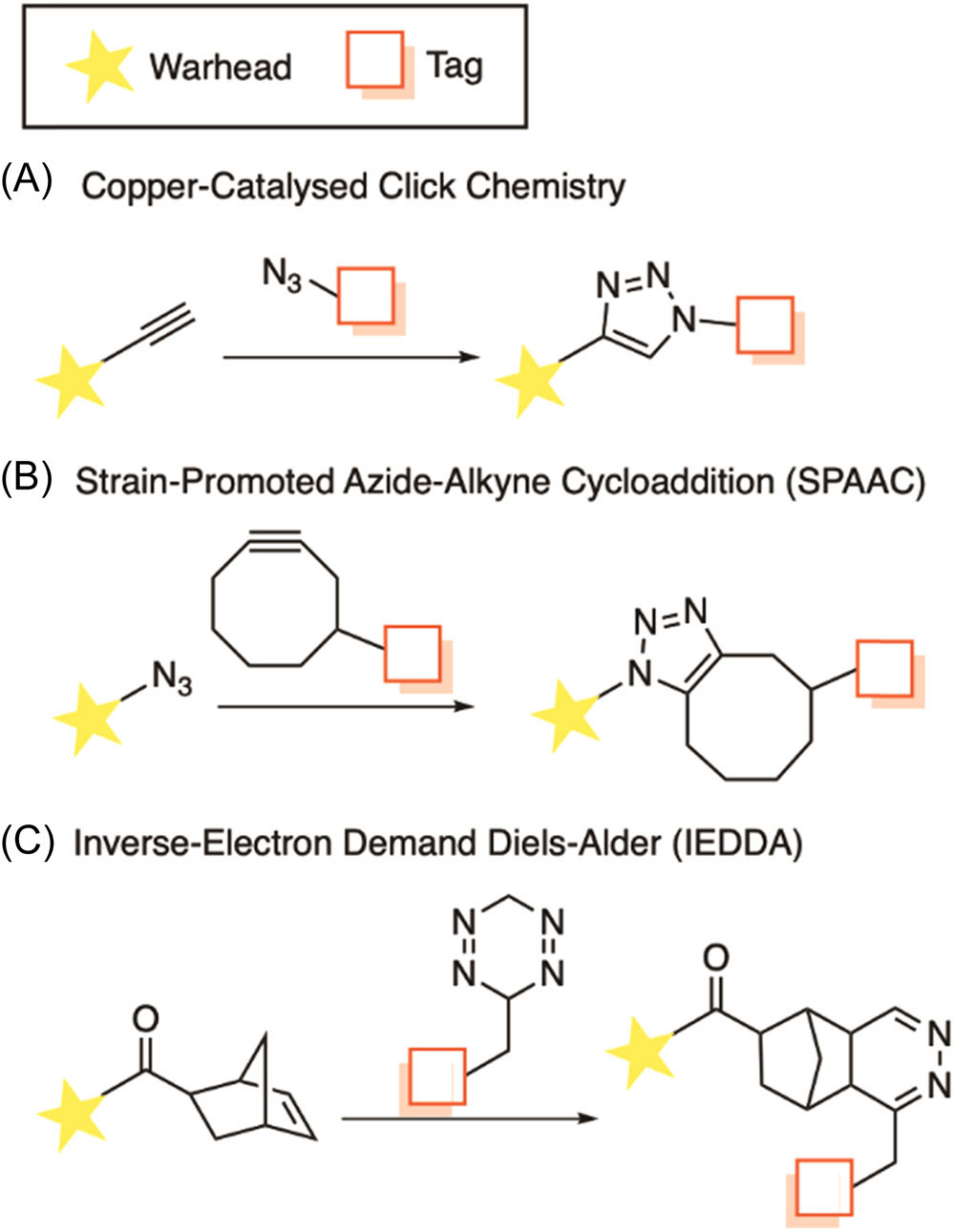
Common bioorthogonal reactions used in the construction of chemical probes. [Color figure can be viewed at wileyonlinelibrary.com]

The copper‐mediated CuAAC reaction uses terminal azide and alkyne functionalities to form a 1,2,3‐triazole linkage based on the Huisgen Cycloaddition (Figure 2A). However, the use of cytotoxic copper reagents can be undesirable and are therefore not applicable for use in live *Plasmodium*.^113^ Therefore, copper‐free methods have come into prominence for this purpose. The first of these is SPAAC, which uses a strained cyclooctene ring to promote the formation of the triazole linkage (Figure 2B).^114^ Other common copper‐free methods also employ facile cycloaddition chemistry, such as IEDDA reactions. An example of this type of reaction employs activated or strained alkenes such as norbornene with a tetrazines functionality (Figure 2C).^115^ Bioorthogonal probes are particularly useful where steric restrictions of target binding preclude conjugation to a larger group in situ and lead to a significant reduction in probe activity.^116^ A functionalized biotin molecule can be conjugated to the click chemistry partner to enable streptavidin affinity capture.^117^ Fluorescent tags can also be conjugated to enable in‐gel fluorescence and live‐cell imaging.^118^ Importantly, the same bioorthogonal probe can be used for both experiments, expanding its utility.

### 3.2 Photo‐affinity based protein profiling

**Figure 3:**
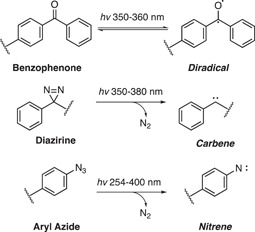
Common photoaffinity ligands. Benzophenones, diazirines, and aryl azides generate highly reactive species upon excitation with UV light which facilitate photocrosslinking to adjacent proteins when included in a probe structure.

A major disadvantage of traditional AfBPPs is that their effectiveness is dependent on the activity or affinity of the probe as well as the abundance of the protein target.^100^ UV‐mediated covalent photo‐crosslinking or photo‐affinity labeling (PAL) has been developed as a method to circumvent this problem.^119^ PAL involves adding a photo‐reactive tag to the probe structure and upon UV irradiation, a reactive radical is generated that allows covalent linkage to proteins in close proximity to the chemical probe—ideally a protein for which the probe has the highest affinity. The photo‐reactive tag consists of groups that can generate reactive diradicals, carbenes, or nitrenes yielded from benzophenones, diazirine, and aryl azide, respectively (Figure 3).^120^ To allow for in‐gel fluorescence or affinity capture, often PAL ligands also feature a second functional tag such as a click chemistry handle or a biotin/streptavidin binding partner.

The choice of PAL handle comes with several caveats. Benzophenones have some distinct biochemical advantages in that they are more chemically stable than the other groups and can be handled in ambient light.^121^ Additionally, the excitation is reversible in the absence of a suitable C‐H bond to insert into, therefore a sample can be repeatedly excited to improve yields.^122^ However, an increase in excitation time can have implications in increasing nonspecific labeling.^123^ The size of the benzophenone group can also be difficult to incorporate into the structure without diminishing affinity.^124^ Therefore, the comparatively small size of aryl diazirine and aryl azide groups has led to an uptick in their usage.^105^ Aryl diazirines produce better photo‐crosslinking yields than aryl azides, perhaps due to the increased reactivity of the carbene over the nitrene.^125^ Benzophenones and aryl diazirines are also maximally activated at relatively high wavelengths, causing minimal damage to proteins.^126^ However, unlike benzophenones, both aryl diazirines and aryl azides can be susceptible to UV‐induced rearrangement and photolysis which reduces the efficiency of labeling.^127^ Nonspecific labeling can be considered a broad problem for all PAL probes as pulldowns are generally performed in great excess.^128^ This becomes particularly problematic where target abundance is low, and nonspecific binding obscures its detection.^129^ Demonstrating a labeling profile that is specific versus a negative control probe and is disrupted by free compound competition is very important to ensure that specific binding.^129^

### 3.3 Affinity based protein profiling examples

AfBPP has been commonly employed for both target identification and target engagement of antimalarials under development. Reliable pull‐down of the target from parasites is typically reliant on having a highly potent and target‐selective compound as a template for the design of the AfBPP in addition to the appropriate controls to exclude promiscuous and abundant proteins. To provide confidence in the pulled‐down protein are indeed genuine, a bioorthogonal technique should be used to provide supporting evidence. Several AfBPP examples are given below that successfully pulldown the target which is confirmed by a target validation method. These examples include AfBPPs based on MMV048, purvalanol B, purfalcamine, and WM382.

#### 3.3.1 Quinoline antimalarials

**Figure 4:**
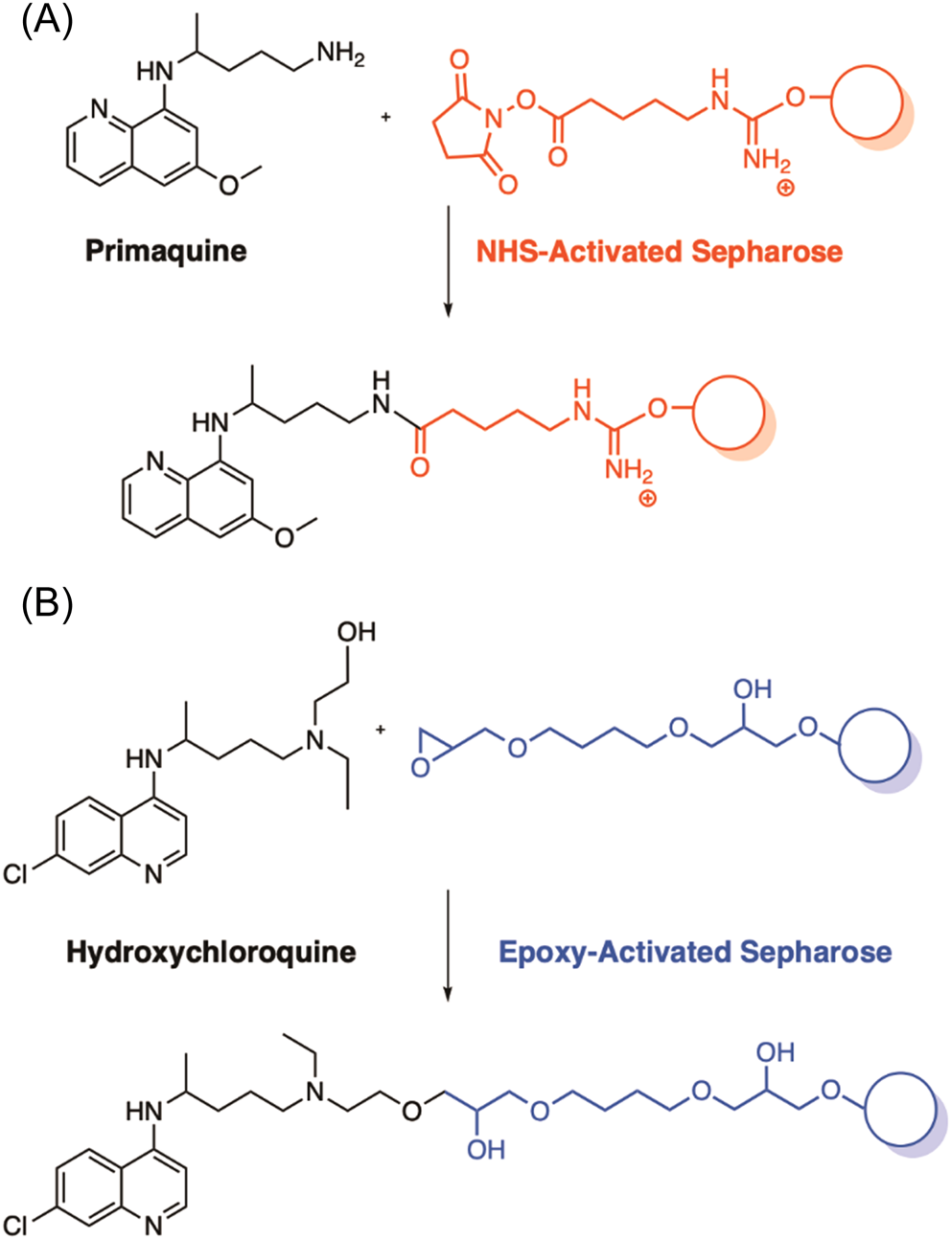
Resin immobilized probes of the known antimalarials primaquine and hydroxychloroquine for the identification of cellular targets. Pulldown of the resin immobilized probes in infected erythrocyte lysate resulted in the enrichment of two human proteins, ALDH1 and QR2. Biochemical validation confirmed QR2 as a probable target and indicated that inhibition of ALDH1 may be the result of an off‐target effect. [Color figure can be viewed at wileyonlinelibrary.com]

The first published use of chemical probes in antimalarial target identification aimed to discover binding or reactive protein targets of quinoline antimalarials. Quinoline antimalarials include hydroxychloroquine (HQ), chloroquine (CQ), primaquine (PQ), and mefloquine (MFQ),^97^ which have been in clinical use since the mid‐20th century without a well‐defined mechanism of action.^130^ Structural similarity between the quinolines and purine nucleotides led to a hypothesis that they may target purine (ATP) interacting proteins.^97^ Therefore, two types of probes were employed, a promiscuous ATP‐Sepharose probe for application in competition experiments as well as quinoline‐Sepharose conjugates. PQ was affixed with its primary amine functionality to NHS‐activated Sepharose (Figure 4A), and HQ with its free hydroxyl group to epoxy‐activated Sepharose (Figure 4B). The ATP‐Sepharose probes were incubated with infected RBC cellular extracts to pull down the RBC and *P. falciparum* purine proteome. Eluting with PQ, CQ, and MFQ did not result in the identification of any *P. falciparum* proteins. However, the drugs were highly selective for two human proteins from RBC extracts, aldehyde dehydrogenase (ALDH1) and quinine oxidoreductase 2 (QR2). The same experiments were performed with the PQ and HQ conjugated probes, again selectively eluting only human proteins ALDH1 and QR2. Subsequent in vitro target validation identified potent inhibition of QR2 by CQ and PQ, and only weak inhibition of ALDH1 by CQ. Together, this implicated human QR2 as a probable target of CQ and PQ, whose role is the detoxification of quinones which can cause oxidative damage.^131^ The malaria parasite itself is sensitive to oxidative damage,^132^ and inhibition of QR2 by quinolines may create an inhospitable environment for parasite growth. While the inhibition of ALDH1 likely does not represent the quinolines' antimalarial target, the authors believe that affinity to ALDH1 may explain chloroquine's reported retinopathy. ALDH1 may have a metabolic role in protecting the eye from UV damage,^133^ and treatment with chloroquine indeed results in the hyperaccumulation of retinaldehyde in the retina.^134^, ^135^, ^136^

#### 3.3.2 MMV048

**Figure 5:**
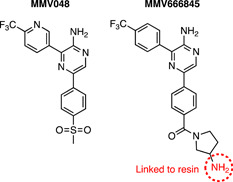
A resin immobilized chemical probe of MMV048 used in the identification of *Plasmodium falciparum* cellular targets. An active analog of MMV048 with an amine functionality was chosen to link to the Sepharose resin. Phosphatidylinositol 4‐kinase (PI4K) was identified as a probable target, confirmed with competition experiments with MMV048 and subsequent in vitro resistance evolution. [Color figure can be viewed at wileyonlinelibrary.com]

The antimalarial candidate, MMV048 (Figure 5)^137^ was developed from a 2‐aminopyridine class identified from a phenotypic high‐throughput screen of the commercial SoftFocus kinase library.^19^ As such the exact molecular target of MMV048 was unknown, although presumed to be a kinase.^137^ For target deconvolution, a related analog MMV666845 was chosen as it possesses a primary amine functionality (Figure 5).^137^ This moiety was covalently immobilized to Sepharose beads by an undisclosed method and was subsequently treated with parasite‐infected RBC lysate. Eluting bound proteins with unlabeled MMV048 identified one high‐affinity binding protein, phosphatidylinositol 4‐kinase (PI4K).^137^ Similar to the quinolones, competitive inhibition with unlabeled MMV048 with “kinobeads” derivatized with a broad set of ATP competitive kinase inhibitors that covered approximately 50% coverage of the *Plasmodium* proteome was also performed, resulting in a dose‐dependent competitive elution of PI4K in the presence of MMV048.^137^ In vitro resistance evolution experiments also identified mutations in PI4K validating it as a target.^137^ More recently, kinobeads and lipid‐kinobeads with coverage of 54 *P. falciparum* kinases were used to uncover that sapanisertib had the strongest competition for PKG (PF3D7_1436600), PI4Kβ (PF3D7_0509800), and PI3K (PF3D7_0515300) using *P. falciparum* lysate.^138^ PfPKG and PfPI4Kβ were confirmed as targets of sapanisertib using an ATP competitive biochemical inhibition using recombinant protein, further demonstrating the utility of kinobeads in target identification of antimalarials with kinase‐like chemotypes.

#### 3.3.3 Torin 2

**Figure 6:**
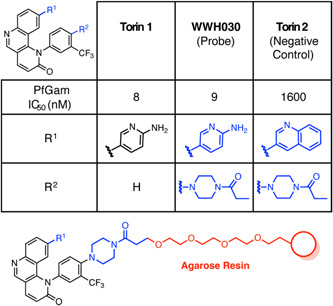
Summary of the human mTOR inhibitor Torin 2 *P. falciparum* activities and AfBPP design. An equipotent and structurally related compound WWH030 was used to construct a chemical probe for Torin 2 as it possessed a suitable handle. The negative control was constructed from the significantly less active Torin 2. Pulldown in *P. falciparum* gametocytes revealed putative targets, including phosphoribosyl pyrophosphate synthetase, aspartate carbamoyltransferase, ATCase, and (PF3D7_0914700). [Color figure can be viewed at wileyonlinelibrary.com]

Torin 2 is a known competitive ATP inhibitor of regulatory the kinase mTOR with indications in the treatment of some cancers.^139^ In a screen of known chemical entities, it was shown to have potent activity against both gametocytes and asexual *P. falciparum*.^140^ Due to the absence of an mTOR homolog within the *P. falciparum* genome, target deconvolution was performed using resin immobilized AfBPP.^140^ Torin 2 lacks a suitable functional group for attachment to the resin, therefore, the analog WWH030 with a piperazine carboxamide moiety which had minimal impact on gametocytocidal activity was used as the AfBPP (Figure 6A). WWH030 was conjugated to NHS Sepharose, along with a structural similar Torin 1 compound with weak parasite activity which was employed as control AfBPP (Figure 6B). Thirty‐one proteins were specifically pulled down with the Torin 2 chemical probe in gametocyte lysate which was complemented by DARTS target identification (discussed later in Section 3.2), identifying 3 common putative targets: phosphoribosylpyrophosphate synthetase (PF3D7_1325100, ribose‐phosphate diphosphokinase), aspartate transcarbamoylase (PF3D7_1344800, PfATC) and a putative transporter (PF3D7_0914700).^140^ PfATC is an enzyme involved with pyrimidine biosynthesis, a pathway targeted directly and indirectly by a number of antimalarials. To validate its role in Torin 2 antimalarial activity, dose–response assays were performed against recombinant PfATC, reported at 68 µM.^141^ Transgenic parasites overexpressing ATC were used to validate Torin 2, which revealed a more than 18‐fold reduction in activity compared to the control.^141^ The other two putative targets have not been further validated to date. However, Torin 2 analogs have since been developed with greater selectivity for parasites over the human mTor enzyme, improved solubility, and metabolic profile.^142^ These analogs have been shown to exert antiparasitic activity through inhibition of phosphatidylinositol 4‐kinase (Pf PI4KIIIβ).^142^

#### 3.3.4 Purvalanol B

**Figure 7:**
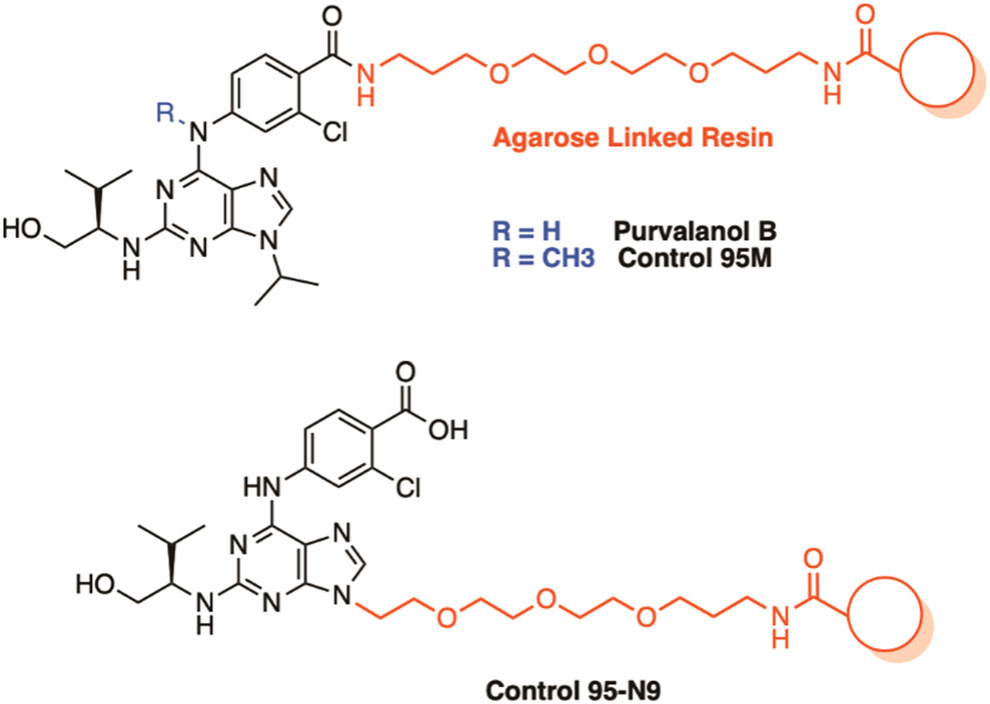
A resin immobilized chemical probe used in the identification of *Plasmodium falciparum* targets of the human cyclin‐dependent kinase 2 (CDK2) inhibitor purvalanol B. *Purvalanol B* and related inactive controls were immobilized via a PEG linker to an agarose resin for target identification in *P. falciparum*. Pulldown identified only one potential target, *P. falciparum* casein kinase 1 (PfCK1). [Color figure can be viewed at wileyonlinelibrary.com]

Purvalanol B was identified from a screen of a known human drug library and subsequently investigated using an AfBPP approach.^143^ The drug is known to target the human cyclin‐dependent kinase 2 (CDK2),^144^ a member of an important family of cell cycle regulators implicated in cancers and neurodegenerative diseases.^145^ However, this compound has also been found to have an antiproliferative effect on a range of human protozoan parasites, including *P. falciparum*.^143^ After examination of the x‐ray structure of purvalanol B in complex with human CDK2, it was established that the carboxylic acid group would make an appropriate handle for functionalization in an AfBPP as it would have minimal effect on binding (Figure 7).^144^ Previous SAR indicated that the addition of a methyl group at the N6 position on analog 95 M significantly diminished CDK2 inhibitory activity so was used as a control in the AfBPP study (Figure 7).^146^ The authors also found that large functionalities at N9 reduced CDK2 inhibition, therefore, they placed the linker at this position for use as an additional control (95‐N9).^146^ Pulldown in *P. falciparum* resulted in a singular protein, casein kinase 1 (CK1). The authors found that purvalanol B did not significantly inhibit mammalian CK1, but potently inhibited *Pf*CK1 (IC_50_ 0.30 μM) despite the high sequence conservation. Unfortunately, this discovery has not resulted in further exploration of a CK1‐targeted antimalarial, however, the study has prompted the investigation of inhibitors in other protozoan parasitic species examined such as *Leishmania* and *Trypanosoma*.^147^, ^148^, ^149^

#### 3.3.5 Purfalcamine

**Figure 8:**
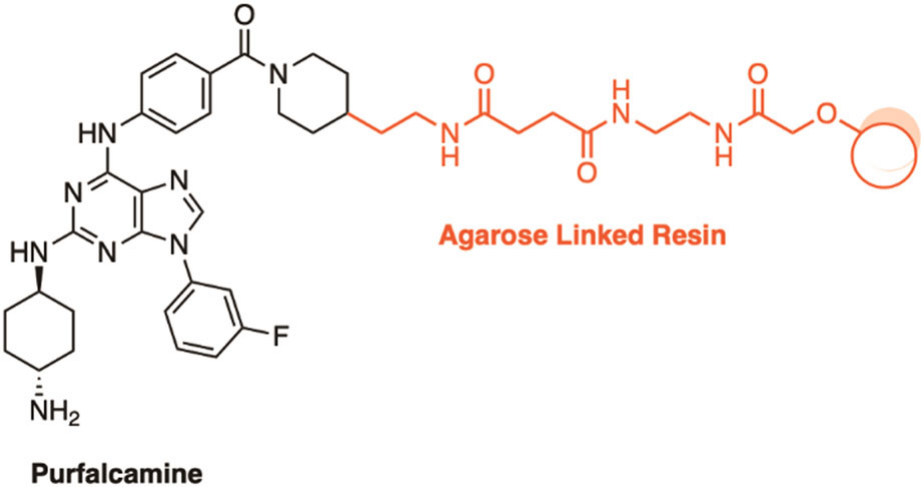
A resin immobilized chemical probe for validation of the cellular targets of purfalcamine. Pulldown with this probe identified several proteins with PfCDPK1 as the likely candidate. [Color figure can be viewed at wileyonlinelibrary.com]

Purfalcamine was identified as a potent inhibitor of *P. falciparum* calcium‐dependent protein kinase 1 (*Pf*CDPK1) from a target‐based screen of a kinase‐directed heterocyclic library.^150^ To validate the parasite targets of purfalcamine, an agarose immobilized purfalcamine AfBPP was incubated with parasite lysate in the absence or presence of unlabeled purfalcamine (Figure 8).^150^ The AfBPP pulled down a hypothetical protein (PF13_01116), a putative FAD‐dependent glycerol‐3‐phosphate dehydrogenase (PFC0275w), a conserved hypothetical protein (PFF0785w), and *Pf*CDPK1.^150^ The highly abundant pyruvate kinase was pulled down in both the competition and noncompetition conditions, therefore was not considered a specific target. Microscopic examination identified that purfalcamine caused cycle arrest at the schizont stage,^150^ consistent with *pfcdpk1* gene transcription supporting *Pf*CDPK1 as the primary target.^151^

#### 3.3.6 Imidazopyridazine antimalarials

**Figure 9:**
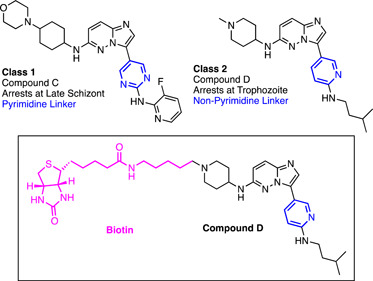
Imidazopyridazine compounds identified using a target‐based screen against PfCDPK1. Two classes of imidazopyridazine compounds were identified, differing in their aromatic linker. Class 1 imidazopyridazines possessed a pyrimidine linker and arrested parasites at the late schizont stage. Class 2 imidazopyridazines are linked via nonpyrimidine aromatic rings and arrest at the trophozoite stage. Biotinylation of compound D enabled streptavidin affinity pulldown for the identification of cellular targets. The probe identified the molecular chaperone PfHSP90 as a potential target for the compound. [Color figure can be viewed at wileyonlinelibrary.com]

The imidazopyridazine antimalarial scaffold was discovered in another target‐based screen against *Pf*CDPK1 employing two different compound libraries.^152^ The first was a library containing a diverse set of 35,422 compounds, and the second was the BioFocus kinase library. This identified a number of scaffolds with sub‐nanomolar inhibitory activity against *Pf*CDPK1, including the imidazopyridazine chemotype. While compounds of this class were indeed active against asexual *P. falciparum* parasites, the subsequent SAR studies indicated that the level of *Pf*CDPK1 inhibition correlated poorly with the inhibition of parasite growth.^153^ Subsequent chemical genetics altering the kinase sensitivity to inhibitors established that inhibition of *Pf*CDPK1 did not alter parasite viability in asexual stages, ruling it out as a potential target.^154^ This also called into question the validity of *Pf*CDPK1 as a legitimate target for the previously mentioned 2,6,9‐purine purfalcamine. Phenotypic studies were therefore initiated on imidazopyridazine analogs, where it was discovered that two sub‐structural imidazopyridazine classes had distinct mechanisms of action depending on their aromatic linker. Compounds with a pyrimidine‐linker arrested parasites at late schizogony, whereas the non‐pyrimidine‐linker arrested parasites at trophozoite stage (Figure 9).^154^

The authors noted the schizontocidal activity of class 1 matched phenotype of a kinase closely related to *Pf*CDPK1, cGMP‐dependent kinase (PKG).^154^ Indeed, the antiparasitic SAR of class 1 compound closely correlated with PKG IC_50_.^154^ Additionally, chemical genetics performed on PKG identified a link between the kinase's sensitivity to the inhibitor and parasite viability.^154^ For target identification of class 2 nonpyrimidine targets, an affinity pulldown approach was taken. Compound D (Figure 9) was conjugated to biotin for affinity capture of targets with streptavidin‐agarose. This pulldown only identified one significant target—HSP90, a molecular chaperone containing an ATP binding site that is essential for mediating the transition from ring to trophozoite development.^155^ Recombinant *Pf*HSP90 binding assays were subsequently used to confirm compound interaction of 6.17 μM, similar to another HSP90 inhibitor 17‐AAG which also blocks parasite development at the trophozoite stage.^154^ The authors considered this to be a promising target, although could not rule out other targets not able to be pulled down in this study. Indeed, a discrepancy between the potent 360 nM cellular activity and weak protein binding points to this possibility. Heat shock proteins are well‐known promiscuous binders as a function of their role in protein folding.^156^ Accordingly, HSP90 is included in the CRAPome, a repository for common nonspecific binders in AfBPP for the human and yeast proteomes.^156^ Without a negative control compound or competition experiment nonspecific interactions cannot be ruled out for this target.

#### 3.3.7 Plasmepsin X

**Figure 10:**
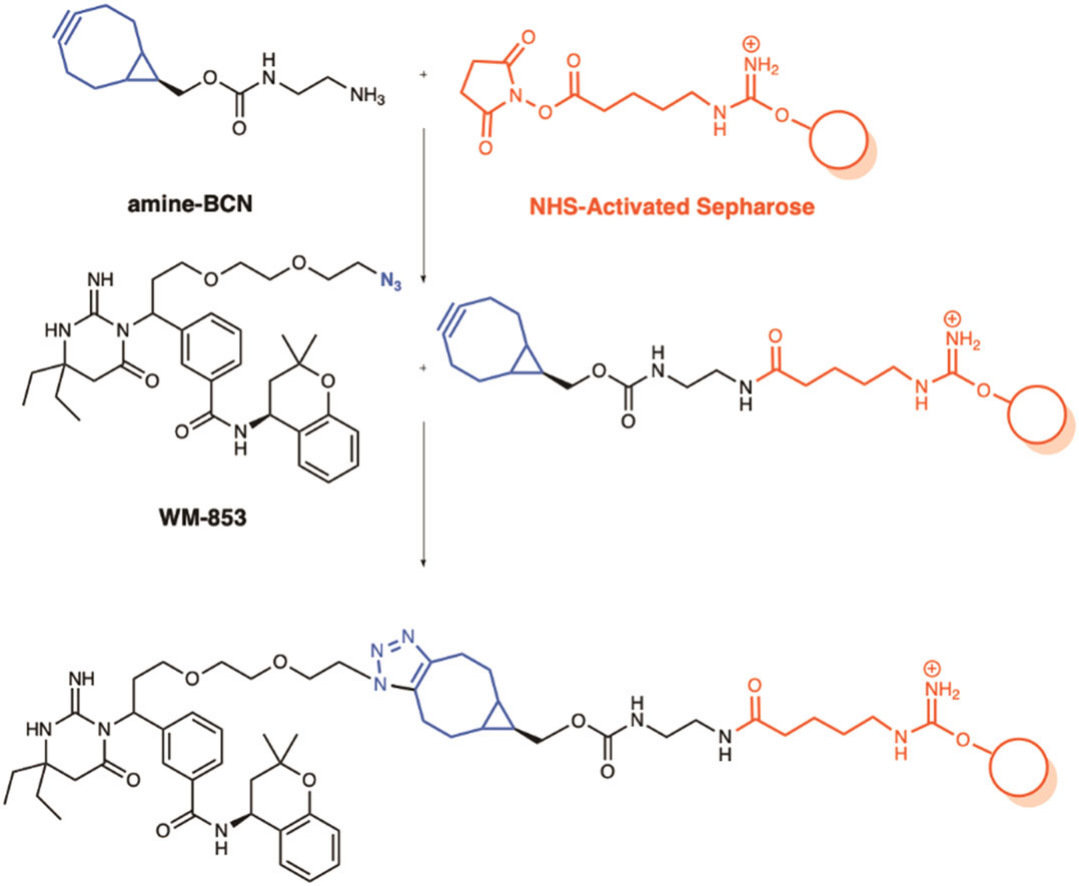
SPAAC probes used in the target validation of WM382 against plasmepsin X. An azide functionalized derivative of the lead compound (WM‐853) was used to attach the compound of interest to a Sepharose resin using SPAAC copper‐free conditions. These probes were incubated with lysate from an HA‐tagged PMX parasite line where PMX was identified as a binder by western blot with an anti‐HA antibody. Pulldown of PMX was competitively inhibited by the addition of the parent compound WM382. [Color figure can be viewed at wileyonlinelibrary.com]

Plasmepsins are aspartic proteases, some of which are essential and are potential drug targets, including plasmepsin IX and X (PMIX and PMX) which are involved in the parasite invasion and egress pathway.^157^, ^158^ Following a high‐throughput screen of an aspartic protease inhibitor library, it was discovered that a novel scaffold inhibited *P. falciparum* growth with nanomolar potency.^159^ By selecting for resistance, PMX was determined as a probable target of these compounds.^159^ An AfBPP approach was implemented to validate PMX as the target of these compounds. First, a hemagglutinin A (HA) tagged PMX parasite line was developed that would be used to detect the target protein by western blot with anti‐HA antibodies.^159^ A click chemistry AfBPP approach was then used to construct the chemical probes (Figure 10). The solid support was first synthesized, attaching the strained cyclooctyne amine‐BCN to NHS‐Sepharose via its terminal amine. Next, the lead active compound (WM382) was modified with an azide moiety by a PEG linker to give the probe called WM853. Copper‐free click chemistry was used to attach these two hemispheres together for the final pulldown. Due to the stage‐specific expression of PMX, the pulldowns were performed using the lysate of late schizont stage saponin‐treated parasites. Western blot identified efficient pulldown of PMX which was interrupted by the presence of free excess lead compound WM382. Interestingly, WM382 also inhibits PMIX at a lower affinity than PMX,^160^ but was not pulled down in this study, highlighting the requirement for high‐affinity ligands for successful pulldown of genuine targets.

### 3.4 Activity based protein profiling examples

ABPPs based on antimalarials that typically covalently engage their protein targets are by their very nature reactive and therefore potentially have several targets rather than one exclusive target. These ABPPs typically pulldown many protein targets, which can be difficult to deconvolute and determine whether each protein is a genuine binding protein. A key example in the following section is the endoperoxide antimalarials which are known to mechanistically cross‐link with many proteins, and therefore using the ABPP method it has been difficult to reliably detect target proteins.

#### 3.4.1 N‐251 and N‐89

**Figure 11:**
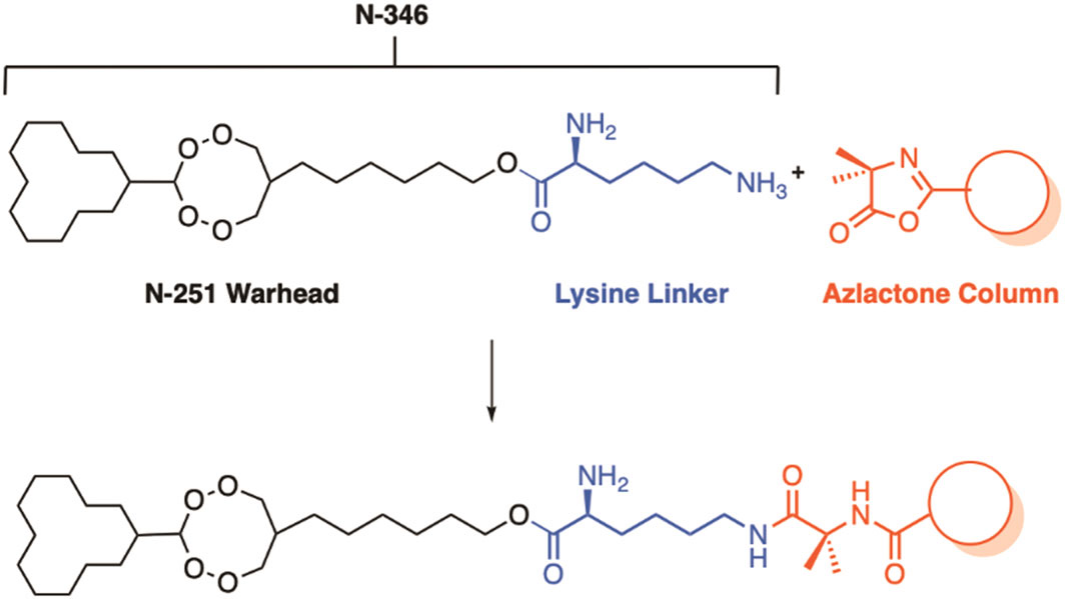
A resin immobilized chemical probe for the novel endoperoxide N‐251. The novel endoperoxide N‐251 was linked to an azlactone Sepharose resin via a lysine linker to create the probe N‐346. Treatment with cellular lysate resulted in the enrichment of PfERC, Pf14‐3‐3, and PfHSP70. Weak binding of N‐251 and N‐89 to PfERC was confirmed subsequently by surface plasmon resonance (SPR). [Color figure can be viewed at wileyonlinelibrary.com]

ABPPs were implemented to identify the targets of novel endoperoxide drugs N‐251 and N‐89.^161^ In the ABPP design, a lysine linker was coupled to the hydroxyl group of N‐251, termed **N‐346**, for conjugation to the resin functionalized with an azlactone (Figure 11).^161^ *Pf*ERC, *Pf*14‐3‐3, and *Pf*HSP70 were the highest enriched proteins from the pulldown with the **N‐346** ABPP. Subsequently, differential protein expression analysis confirmed that the expression of these proteins was altered by treatment with N‐251 and N‐89.^161^ The latter two are unlikely targets as they are known to promiscuously bind compounds in their roles as kinase regulator and molecular chaperone, respectively.^162^ *Pf*ERC is an essential ER‐resident protein, important for asexual parasite egress.^163^ N‐251 and ‐89, but not the related endoperoxide, artemisinin, were subsequently confirmed to bind weakly to *Pf*ERC by surface plasmon resonance (*K*_D_ 1.6 × 10^–4^ M and 3.8 × 10^–3^ M).^161^ The binding of these compounds may represent a mechanism for these novel endoperoxides or may be the result of nonspecific binding.

#### 3.4.2 Artemisinin

**Figure 12:**
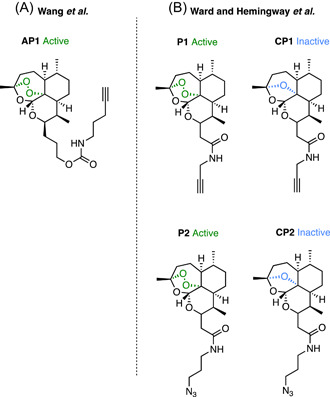
Artemisinin‐based click chemistry probes. (A) Alkyne and azide click chemistry probes by Hemingway and Ward et al. identified 42 common proteins in an affinity pulldown, with a majority containing a glutathione binding motif which may be particularly susceptible to radical alkylation. When **P1** was retested by Maser et al. with additional controls far fewer proteins were pulled down, none of which were found in the original study. (B) Alkyne probe **AP1** pulled down 125 high‐confidence proteins with similar pathway coverage to the **P1** and **P2** probes. [Color figure can be viewed at wileyonlinelibrary.com]

Two concurrent seminal studies on the mechanism of Artemisinin by Wang et al.^164^ and Ward and Hemingway et al.^165^ utilized ABPPs with click chemistry handles. This method allowed for the in situ use of the probes which is important given the evidence of site‐specific activation of the endoperoxide moiety.^166^ Both studies attached the click chemistry handles via a linker to the C10 position of the ART structure which proved not to significantly reduce antiparasitic activity.^164^, ^165^ Differences in linker structure and size separate the two studies (Figure 12), where Ward and Hemingway et al. denote their probes as **P1** and **P2** while Wang et al. denote their probe as **AP1**. **AP1** features a much longer carbamate linker, terminating in an alkyne click chemistry handle (Figure 12A).^164^ **P1** and **P2** feature a short amide linker, deliberately chosen by the authors to avoid issues with cytotoxicity that have been reported with longer amide linkers (Figure 12B).^167^ Both alkyne and azide handles were conjugated for comparison of copper‐mediated and copper‐free click conditions due to previous studies proposing the potential for copper to activate artemisinin in a similar manner to iron in hemozoin.^168^ A final difference between the two studies is the use of inactive control ABPPs by Ward and Hemingway et al. who constructed corresponding non‐peroxidic probes **CP1** and **CP2** which were inactive against parasites. Despite these differences, the workflow of the two studies was similar. Live parasites were exposed to the probes to allow for protein alkylation and proteins were subsequently extracted. The extracts were combined with a clickable functional group, either a fluorophore for in‐gel fluorescence analysis or biotin conjugate for streptavidin bead affinity capture. **P1**, **P2**, and **AP1** all alkylated a large number of proteins by in‐gel fluorescence.^164^, ^165^ Control probes **CP1** and **CP2** did not show any alkylation by in‐gel fluorescence.^165^ The authors found **AP1** alkylation to be dose‐dependent, unobservable in uninfected erythrocytes, and antagonized by co‐incubation with free radical scavengers.^164^ This supports the parasite‐specific activation of the scaffold and the formation of a free radical.

When coupled to the streptavidin beads, probes **P1**, **P2**, and **AP1** similarly pulled down a wide range of targets involved in many essential pathways.^164^, ^165^ **P1** identified 58 high‐confidence proteins, four of which were pulled down nonspecifically in low abundance by the inactive **CP1** probe.^165^ **P2** pulled down 62 proteins, 42 of which were in common with **P1**,^165^ while the control azide probe **CP2** did not pull down any protein nonspecifically.^165^ The copper‐free method with **P2** appeared to detect these proteins with greater sensitivity due to the high efficiency of the strain‐promoted click reaction with the DIBO cyclooctyne.^165^ **P2** was also assessed with cell lysate and showed no significant difference in the labeling of proteins.^165^ **AP1**, on the other hand, pulled down high confidence 124 protein targets, including a further 125 proteins pulled down in repeat experiments.^164^ It has been suggested that the larger range of identified targets is due to the increase in linker size and thus lipophilicity.^165^ This cannot be assessed without the comparison of an inactive control for **AP1**.

The overall coverage of parasite pathways between the ABPP types appears to be similar. Alkylated proteins converge on a subset of pathways, including glycolysis, nucleic acid biosynthesis, protein biosynthesis, invasion, protein transport, and redox antioxidant defense.^164^, ^165^ Notably, a substantial number of the proteases involved in hemoglobin digestive pathway in the DV were labeled, including plasmepsin I, plasmepsin II, and cathepsin D.^164^, ^165^ However, the incomplete labeling of proteases in this pathway (e.g., falcipain II and falcipain III) suggests a degree of selectivity to ART‐protein alkylation.^164^, ^165^ Analysis of targets pulled down by **P1** and **P2** indicated a correlation with proteins that had a glutathione binding motif.^164^ The authors suggested this free thiol may be an easy target for the ART free radical which has previously been shown to form cysteine adducts.^169^ The formation of these adducts may directly contribute to the specificity seen above to aspartic and cysteine proteases in the DV hemoglobin digestion pathway.

Interestingly, alkylated targets of **P1** and **P2** were shown to be differentially affected by the addition of the iron chelator, DFO. The plasmepsins and the majority of the glycolytic enzymes were not significantly affected by DFO treatment, whereas ornithine aminotransferase (PfOAT) was.^165^ PfOAT was also identified as a target of **AP1**, and in vitro biochemical analysis showed that binding occurred only in the presence of added haemin.^164^ This binding was further enhanced by the addition of reagents that reduced hemin to heme (Vitamin C, GSH, and Na_2_S_2_O_4_).^164^ The addition of ferrous iron, on the other hand, had no impact on the binding of **AP1** to PfOAT.^164^ Additionally, the binding of **AP1** to PfOAT appeared to be protein structure‐dependent as heat denaturation of PfOAT diminished binding.^164^

Both studies also explored the mechanisms of ART activation. Ward and Hemingway et al. tested the effect of DFO pre‐treated cellular homogenates on **P1** alkylation and found that it significantly reduced but not ablated pulled down proteins.^165^ This data suggested that ART may be involved in a nonferrous iron‐mediated activation pathway, although this notion was questionable as the concentration of DFO used for chelation in live parasites and in free heme homogenates were significantly different.^170^ Wang et al. then tested the effect of iron chelating agents DFO and DFP (deferiprone) in live parasites which did not result in a significant reduction in **AP1** protein alkylation by in‐gel fluorescence.^164^ However, a cysteine protease inhibitor N‐acetyl‐Leu‐Leu‐Norleu‐al (ALLN) that inhibits the production of heme via the parasite hemoglobin digestion pathway,^171^ caused a significant decrease in the fluorescent labeling of proteins by **AP1**. Together, this points to heme as the predominant source of ART activation.^164^ However, this fails to explain the activity of ART in the early ring stage, where hemoglobin digestion does not occur.^172^ The authors surmised that hemoglobin biosynthesis that occurs at this stage could be a source of heme for ART activation. To test this, synchronized early ring parasites were pretreated with the hemoglobin synthesis inhibitor SA which proved to reduce the level of ART protein binding by **AP1**.^164^ Hemoglobin digestion inhibitor ALLN also had no effect on **AP1** alkylation in ring stages.^164^

Maser et al.^173^ later re‐evaluated the same probes from Ward and Hemingway et al.^165^ (**P1** and **CP1**, Figure 12B) with additional controls. The authors included a DMSO‐treated control, an ART‐treated control, the nonperoxidic control **CP1** as well as multiple probe concentrations.^173^ Remarkably, the proteins alkylated by this experiment had little in common with the targets identified by Ward and Hemmingway et al.^165^ At a concentration of 100 ng/mL **P1** alkylated 15 specific proteins, none of which were present in the original study.^173^ 1000 ng/mL **P1** alkylated an additional eight unique proteins, only one of which was identified in the original study.^173^ The targets identified by Maser et al. were more analogous to Wang et al.^164^ with **AP1** with 10 and 6 proteins in common for 100 and 1000 ng/mL concentrations of **P1**, respectively.^173^ Some targets identified by the previous studies, such as DHFR, were identified in the negative controls of this study and therefore were removed from consideration.^173^ The authors concluded that this variation in ART alkylation is the result of a stochastic binding pattern that may be more linked to radical proximity rather than any specificity.^173^

What is clear from these studies is that the vast number of targets alkylated by ART contribute to its parasite lethality. Glutathionylated proteins appear to represent a large proportion of these targets, presumably due to their susceptibility to alkylation. The peroxide bond is responsible for its activity which is the site of free radical formation. ART also accumulates specifically in infected erythrocytes where it appears heme is responsible for the majority of its activation in later parasite stages. A limitation of these studies is that they do not explore potential noncovalent targets of ART, nor potential nonprotein targets such as heme.^173^ It is also evident that the structure of the probes can vastly affect the results of the pulldowns. This highlights the importance of careful probe design and confirmation of potential targets with other means of target identification or biochemical analysis.

#### 3.4.3 1,2,4‐Trioxolanes

**Figure 13:**
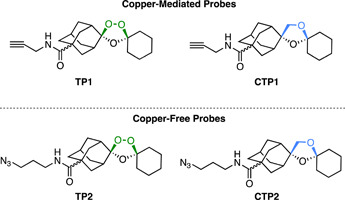
Structures of ozonide click‐chemistry probes TP1 and TP2 and their inactive nonperoxidic control compounds CTP1 and CPT2 synthesized by O'Neill et al. Probes based on an alkyne handle (above) were optimized for a copper‐mediated click reaction, whereas probes with an azide handle (below) use copper‐free methods. 53 common proteins were identified between the two probes with diverse roles, although the majority were glutathionylated. [Color figure can be viewed at wileyonlinelibrary.com]

Based on the rational design of ART‐based probes,^165^ synthetic endoperoxide 1,2,4‐trioxolane ABPPs were constructed.^174^ The probes were designed with minimal linker size and thus lower lipophilicity on the basis of greater specificity and pharmacological relevance.^174^ Both alkyne (**TP1**) and azide (**TP2**) functionalities were used to assess the utility of copper‐mediated and copper‐free methods (Figure 13). Finally, non‐peroxide probes were again synthesized as inactive controls (**CPT1** and **CPT2**; Figure 13). To assess the specificity of the probes, in‐gel fluorescence was determined by clicking on an Alexa Fluor 488 tag.^174^ As was previously observed with the ART probes, the azide probe **TP2** had greater labeling intensity due to the efficiency of the copper‐free strain‐promoted cycloaddition reaction.^165^ The protein alkylation profile of **TP2** was then compared against the analogous ART probe **P2** (Figure 12).^165^ The results of the affinity purification were overwhelmingly similar between the two chemotypes. Of 62 total pulled down proteins, 53 of these were identical.^174^ The roles of these proteins were again in heme digestion, energy supply, DNA synthesis, and antioxidant defense systems.^174^ Interestingly, 70% of the proteins identified were glutathionylated, supporting the theory that the radical formed by heme activation reacts with the disulfide bond present at the site of this posttranslational modification. There were six proteins identified that appeared in one experimental replicate but not the other,^164^ demonstrating the importance of experimental design in ABPP studies.

**Figure 14:**
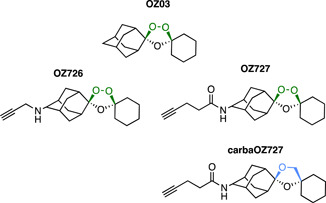
Structures of bioorthogonal ozonide probes by Maser et al. The alkyne‐based copper click chemistry probes identified stochastically alkylated targets with little overlap between similarly structured probes **OZ726** and **OZ727**. [Color figure can be viewed at wileyonlinelibrary.com]

Alongside the re‐evaluation of the ART probe **P1** (Figure 12), Maser et al. constructed novel alkyne functionalized 1,2,4‐trioxolanes (**OZ726** and **OZ727**, Figure 14).^173^ Stringent controls were used including DMSO pretreatment, parent compound **OZ03** pretreatment and the use of a non‐peroxidic control **carbaOZ727**. Interestingly, the degree of overlap between the targets of **OZ726** and **OZ727** was only 30% (6 of 20 proteins).^173^ Indeed, there was no overlap between the proteins alkylated in this study to those of **TP1** and **TP2** (Figure 13).^174^ This again illustrates how influential the structure of probes can be to the target profile. In direct comparison with the ART probe **P1**, the overlap in specificity was just 17% and 13% for **OZ726** and **OZ727**, respectively.^173^ When tested at a 10‐fold higher concentration, **OZ726** alkylated 9 of the 11 proteins identified by **P1** at the same concentration.^173^ However, an additional 16 proteins were identified by **OZ726** at this concentration that were not identified in any of the previous experiments.^173^ As the authors concluded with **P1**, the alkylation of 1,2,4‐trioxolanes appears to be random, which is consistent with the irregular cellular localization of 1,2,4‐trioxolane fluorescent probes.^170^, ^175^, ^176^

#### 3.4.4 Salinipostin A

**Figure 15:**
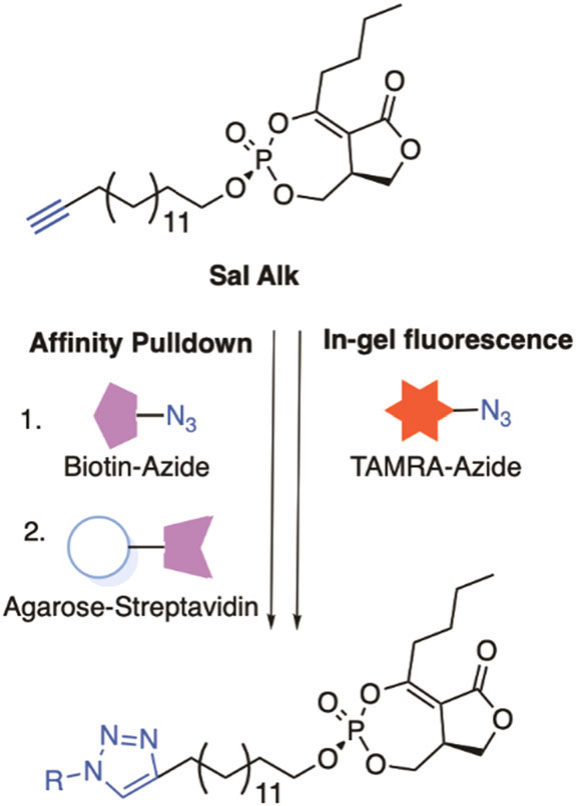
Multi‐functional click chemistry probes of Marine natural product Salinipostin A (Sal A). This multi‐functional probe helped to identify 10 enriched proteins with a common α/β serine hydrolase domain, 4 of which were found to be essential for parasite survival. [Color figure can be viewed at wileyonlinelibrary.com]

Salinipostin A (Sal A) is a marine natural product with low nanomolar activity against *P. falciparum* and has an unknown mechanism of action. Previous mechanistic studies had been unable to generate resistant parasites which suggests that the compound may act through multiple essential pathways.^177^ An alkyne tag was added to a Salinipostin A analog, **Sal alk**, to enable a range of functionalization for ABPP and fluorescence co‐localization studies. (Figure 15).^178^ First, a TAMRA fluorescent label, azide was conjugated to **Sal alk** via click chemistry and upon treatment with parasites confirmed that multiple targets bound to the structure by in‐gel fluorescence.^178^ The labeling of many of these proteins was completed with the addition of unlabeled Sal A in a dose‐dependent manner.^178^ A biotin azide was also conjugated to the alkyne handle for pulldown streptavidin‐resin using parasite lysate pre‐incubated with Sal A or vehicle control. The 10 proteins most highly enriched in these experiments, all possessed classical α/β serine hydrolase domains (Ser‐His‐Asp catalytic triad or a Ser‐Asp dyad).^178^ *piggyBac* mutagenesis studies have determined that four of these are essential for parasite viability,^179^ although have not yet been confirmed as genuine binders in subsequent studies.

#### 3.4.5 Myr‐CoA

**Figure 16:**
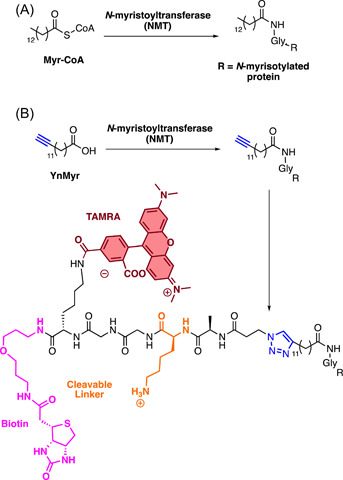
YnMyr probe developed for the recognition of *P. falciparum* N‐myristoylated protein targets. An analog of the MyrCoA with an alkyne handle (YnMyr) was constructed for capture with a trifunctional capture reagent. The terminal azide reagent contains a TAMRA fluorophore for in‐gel fluorescence, a biotin moiety for affinity capture, and a trypsin cleavable linker capable of acting as a tag for the identification of myristoylated proteins by tandem mass spectrometry (MS/MS). [Color figure can be viewed at wileyonlinelibrary.com]

The *Plasmodium N*‐Myristoyl Transferase (NMT) catalyzes the attachment of a myristate lipid tail from Myristoyl‐Coenzyme A (Myr‐CoA) to N‐terminal glycine on specific substrates in membrane trafficking (Figure 16A).^180^ Despite its utility as a target in fungal and trypanosome infections, the genetic essentiality of NMT in *P. falciparum* had not yet been demonstrated.^181^, ^182^ Therefore, ABPPs were designed for use in an NMT substrate capture experiment.^183^ The probe was constructed based on the structure of the enzyme substrate, Myr‐CoA, with an alkyne handle termed YnMyr‐CoA (Figure 16B).^183^ A trifunctional capture reagent was also synthesized featuring a TAMRA fluorescent reporter, biotin affinity capture moiety, and a trypsin cleavable linker (Figure 16B). The cleavable linker allowed the specific identification of the site at which proteins were *N*‐myristoylated without external labeling, resulting in an unambiguous hydrophilic zwitterionic moiety that can be detected with tandem mass spectrometry (MS/MS).^183^ In‐gel imaging was employed to demonstrate that peptide tagging was dose‐dependent which could be competitively inhibited by excess free myristate. The pulldown experiments with avidin purification identified over 30 NMT substrates that have diverse functions including motility, protein transport, parasite development, and phosphorylation pathways. These included *N*‐myristoylated proteins that had been genetically validated for essentiality in other eukaryotes but not in *P. falciparum*. The wide diversity of the pulled down proteins identifies NMT as a promising drug target in *P. falciparum*.

### 3.5 Photo‐crosslinking probe examples

Photo‐crosslinking is generally introduced to an AfBPP or an ABPP to facilitate the covalent linkage of the probe with protein target(s) in parasites. This strategy, followed by a bioorthogonal method to validate the target, has been successfully used by several groups including examples based on the HEA class of protease inhibitors.

#### 3.5.1 ACT‐186128

**Figure 17:**
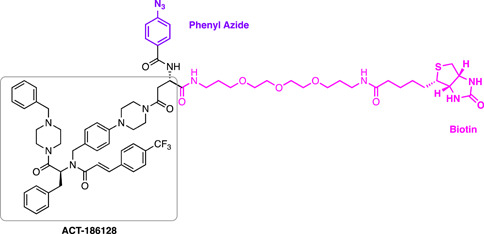
ACT‐186128 chemical probe. [Color figure can be viewed at wileyonlinelibrary.com]

Photo‐crosslinking probes were also used to identify the target of the novel antimalarial ACT‐186128 discovered in a phenotypic screen.^184^ A photo‐AfBPP was developed for application in live cells employing a phenyl azide photo‐crosslinking moiety and a biotin tag for both fluorescent labeling and affinity purification (Figure 17).^185^ Live‐cell imaging enabled by the association of the Alexa488‐streptavidin fluorescent reporter with the biotin moiety showed localization throughout the cytoplasm in all parasite life stages, consistent with its lack of stage specificity.^185^ Pulldown with streptavidin beads was performed after treatment of both intact parasitized red blood cells and saponin‐liberated parasites with the photo‐AfBPP. The pulldown with intact infected RBCs identified one target, the *Pf* multidrug resistance protein 1 (*Pf*MDR1).^185^ The pulldown with saponin‐isolated parasites identified over 20 targets with the highest enriched candidates being *Pf*MDR1, Equilibrative Nucleoside Transporter (*Pf*ENT4), hexose transporter, glideosome‐associated protein 50/secreted acidic phosphatase, and S‐adenosylmethionine synthetase.^185^ The latter five were subsequently ruled out in biochemical validation studies, while *Pf*MDR1 remained a viable target.^185^

#### 3.5.2 Aspartyl protease inhibitors

**Figure 18:**
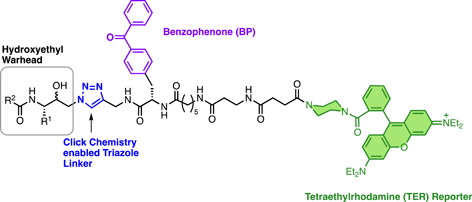
Structure of multifunctional hydroxyethyl chemical probes used for the target identification. Pulldown identified all four known plasmepsins (I–IV) as targets for the hydroxyethyl warhead. [Color figure can be viewed at wileyonlinelibrary.com]

Hydroxyethyl amine (HEA)‐based inhibitors have been used to target aspartyl proteases in the Plasmodium parasite^157^, ^186^, ^187^ and were designed as a non‐cleavable transition state mimic for the functional profiling and identification of plasmepsins.^188^ At the time, just 5 of a putative 10 plasmepsins (PMs) had been identified in *P. falciparum*, the digestive vacuole PMs I‐IV (which are known to be non‐essential and redundant in function)^189^ and plasmepsin V (which is essential for protein export to the host erythrocyte).^190^, ^191^ To validate that these HEA inhibitors genuinely bind to PMs, photo‐AfBPPs were developed possessing an azide click chemistry handle, benzophenone photo‐crosslinking group, and a tetramethylrhodamine (TER) fluorescent reporter (Figure 18).^188^ The TER reporter enabled in‐gel fluorescent quantification and target binding was demonstrated with recombinant protein.^188^ Exposure of the probes to parasite homogenates followed by two‐dimensional (2D) gel electrophoresis and western blot analysis demonstrated that the probe bound to all four digestive PMs.^188^

#### 3.5.3 Signal peptidase inhibitors

**Figure 19:**
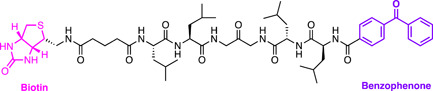
Structure of multifunctional (Z‐LL)_2_ probe used for the target validation study. A benzophenone moiety enabled photoaffinity labeling, while the biotin moiety enabled affinity pulldown which could be detected via western blot for PfSPP. [Color figure can be viewed at wileyonlinelibrary.com]

*P. falciparum* signal peptide peptidase (*Pf*SPP) is an intra‐membrane aspartyl protease located within the parasite endoplasmic reticulum, responsible for the processing of membrane‐embedded signal peptides left behind by the secretory pathway.^192^ *Pf*SPP was hypothesized as a potential target for antimalarial therapy due to the sensitivity of *P. falciparum* to known human SPP and related aspartyl protease inhibitors such as (Z‐LL)_2_, LY‐411575, NITD679, and NITD731.^193^ To validate SPP as the target protein of these inhibitors in *P. falciparum*, a multifunctional AfBPP was synthesized based on the peptidomimetic inhibitor (Z‐LL)_2_ (Figure 19).^193^ The probe featured a biotin tag for affinity purification and a benzophenone moiety to facilitate covalent photo‐crosslinking. Photo‐labeling and affinity purification performed with parasite lysate successfully identified *Pf*SPP binding via western blot analysis using anti‐*Pf*SPP for detection.^193^ Lysate pretreated with free (Z‐LL)_2_, LY‐411575, NITD679, and NITD731, and all demonstrated a competitive reduction in *Pf*SPP pulldown by the probe.^193^ Together, this validated that the known inhibitors of human SPP also targeted the *Plasmodium* homolog.^193^

#### 3.5.4 Albitiazolium

**Figure 20:**
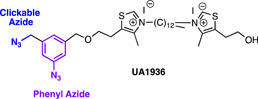
Structure of Albitiazolium bifunctional probe. Photo‐crosslinking and click chemistry affinity purification resulted in the identification of choline/ethanolamine phosphotransferase (CEPT) as a promising target. [Color figure can be viewed at wileyonlinelibrary.com]

De novo phospholipid synthesis is an essential process for the growth and survival of *Plasmodium* parasites.^194^ Therefore, the pathway has generated interest as a promising novel target for antimalarial chemotherapy. The primary phospholipid in *P. falciparum* membranes is phosphatidylcholine which consists of a choline phosphate head group that contains a quaternary ammonium moiety.^194^ A series of highly potent antimalarial choline mimics, the bis‐thiazoliums, were developed based on the ability of quaternary ammonium salts to inhibit phospholipid metabolism.^195^ Unfortunately, the lead compound stemming from this campaign, Albitiazolium,^196^ has since been discontinued in Phase II pediatric trials due to a lack of efficacy.^197^ Before this, its exact mechanism of action had been in question but was primarily considered to be impairing choline transport from the plasma.^198^ Therefore, a bifunctional chemical probe **UA1936** was developed featuring a phenyl azide moiety for covalent photo‐crosslinking as well as a benzyl azido which could be used as a clickable handle for affinity purification and fluorescent labeling (Figure 20).^199^ An inactive AfBPP control, **UA2050**, was also included.^199^ In live cells, fluorescent labeling using click chemistry with the benzyl azido group showed partial colocalization with ER and Golgi‐specific antibodies.^199^ Pulldown was enabled with an alkyne agarose resin after incubation of the probe in whole saponin‐liberated parasites. These parasites were pretreated with either vehicle control or free Albitiazolium.^199^ Two proteins were specifically enriched by the **UA1936** AfBPP. One of these proteins is choline/ethanolamine phosphotransferase (CEPT) which performs the final step in phosphatidylcholine and phosphatidylethanolamine biosynthesis. This was unsurprising as Albitiazolium was previously found to inhibit CEPT activity.^198^ The other is a protein (PFL1815c) with an uncharacterized function. Only CEPT was competitively displaced by treatment with Albitiazolium, confirming it as the target of this antimalarial compound class.^199^

#### 3.5.5 Diaminoquinazoline

**Figure 21:**
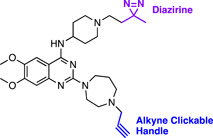
Structure of BIX‐01294 probe. 104 enriched protein targets were identified following photo‐crosslinking and affinity purification, only 35 of which were found to be essential. These targets included those with roles in translational and transcriptional regulation. Notably, histone lysine methyl transferases were absent from the list of targets, which are commonly inhibited by diaminoquinazoline compounds in humans.

Diaminoquinazoline compounds are known inhibitors of human histone lysine methyltransferases (HKMT) and were pursued as potential epigenetic regulators of cancer.^200^, ^201^, ^202^, ^203^, ^204^, ^205^ The scaffold was found to have antimalarial activity in a large screen by GSK and subsequently included in the Tres Cantos Antimalarial Set (TCAMS).^9^ Since then, the scaffold has been extensively optimized and found to have multistage activity.^206^, ^207^, ^208^, ^209^ Despite this, the precise targets of the scaffold remain unclear. There are 10 putative *P*HKMT enzymes but only one, PfSET7, has been successfully purified for biochemical analysis.^210^ To unbiasedly detect targets of **BIX‐01294**, whose SAR had previously been outlined by the group (Figure 21),^208^, ^209^ a photo‐crosslinking chemical probe was constructed by Fuchter et al.^211^ A diazirine photo‐crosslinking group was chosen due to its small size, as well as an alkyne click chemistry handle for functional analysis. A TAMRA azide (AzT) was ligated using click chemistry for in‐gel fluorescent characterization and demonstrated a competitive profile with the parent **BIX‐01294** compound. A TAMRA biotin azide (AzTB) was also used for affinity purification which identified 104 significantly enriched proteins that were filtered through essentiality screening. Only three of these have been found to be essential in *P. falciparum*: PfnPrx which is involved in reversing DNA damage,^212^ NAPL which is a nucleosome assembly protein,^213^ and PfHSP110c which is a cytosolic heat shock protein that prevents the aggregation of asparagine‐rich proteins at febrile temperatures.^214^ In *P. berghei*, which is significantly better characterized, 35 of the enriched proteins have been found to be essential. Of these, the most significantly enriched proteins had roles in translational and transcriptional regulation. Histone lysine methyltransferases were absent from the list, although this may reflect a bias for cytosolic proteins in these lysate‐based experiments, as well as inherent instability or a low abundance of these proteins.

## 4 STABILITY‐BASED METHODS

### 4.1 Cellular thermal shift assay

A recently adapted method of antimalarial target identification and validation is the cellular thermal shift assay (CETSA).^215^ CETSA establishes the target engagement of small molecules in cells or tissues by leveraging an increase in protein thermal stability when bound to a ligand.^216^ At elevated temperatures, proteins begin to unfold, exposing hydrophobic residues, and precipitate out of solution. However, ligand binding leads to an increase in protein stability and remain in solution at higher temperatures.^217^ CETSA has traditionally been used as a target validation technique where stabilized proteins are detected via western blot (CETSA‐WB).^218^ When CETSA is combined with mass spectrometry (CETSA‐MS), it becomes applicable for unbiased, proteome‐wide target identification.^219^ A major advantage of CETSA over other techniques is the ability to verify and quantify the binding of high‐affinity targets in live or lysed cells.

**Figure 22:**
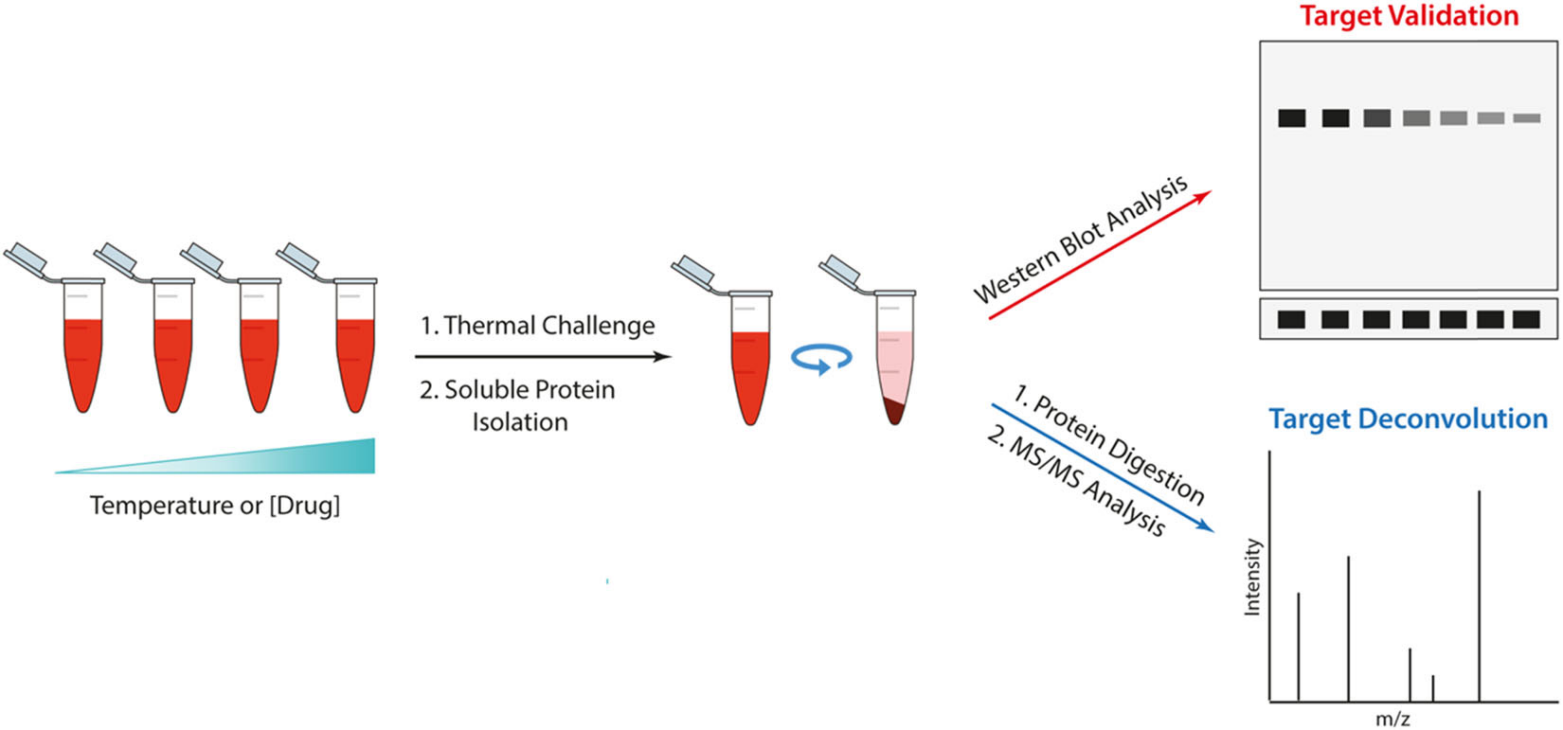
Experimental workflow for CETSA‐MS. Thermal challenge is applied to the samples of interest, modifying either temperature or drug concentration between samples. The soluble protein fraction is isolated, digested, and analyzed by MS/MS or western blot. [Color figure can be viewed at wileyonlinelibrary.com]

The workflow for CETSA‐MS has several key steps (Figure 22). First, the melting behavior of the proteome must be characterized to identify a suitable temperature range for testing. Samples of whole cells, tissues, or lysate are exposed to a gradient of either temperature in the melt curve method or drug concentration in the isothermal drug response (ITDR) method.^220^ Following the thermal challenge, whole cells and tissues are lysed and the soluble protein fraction is isolated.^220^ Proteins from this fraction can be labeled for quantitative determination and then digested into peptide fragments for analysis by tandem mass spectrometry.^219^

While CETSA is a robust method for a wide range of protein targets, not all proteins are amenable to the technique. In general soluble cellular proteins can be easily evaluated through CETSA, however, thermodynamic stabilization is less significant in transmembrane proteins.^220^ Approximately 30% of the plasmodium genome is predicted to have at least one transmembrane domain (PlasmoDB).^221^ Examples with membrane proteins have been reported but require treatment with detergent to first liberate the proteins.^217^, ^222^ Additionally, the nature of the protein‐ligand interaction can influence a lack of stabilization. Should a ligand bind to a domain that is not significantly affected by denaturation or exert its effects by modulating interaction with a secondary protein, a stabilization effect will not be seen.^220^ Increases in thermal stability may not always be a result of direct binding. Proteins involved with complex metabolic pathways can be stabilized by increases in physiological ligands or proteins as a result of drug treatment.^216^ Comparison of CETSA performed with whole‐cells and lysate can be used to control for this factor.^223^ CETSA, such as AfBBPs and ABBPs, are prone to false positives. The use of high and nonphysiological concentrations of the compound can result in the detection of false‐positive binding proteins that may not be involved in the antimalarial mechanism of action. Careful selection of the compound concentration and the use of a structurally similar inactive control compound is helpful in decreasing the number of proteins detected and excluding false‐positive or nonphysiologically relevant proteins.

#### 4.1.1 CETSA examples

##### Quinine, mefloquine, and pyrimethamine

**Figure 23:**
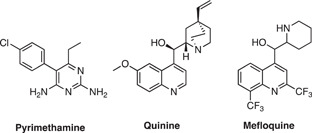
Chemical structures of antimalarials assessed by CETSA. Pyrimethamine, quinine, and mefloquine were used as examples to develop and validate CETSA‐MS as a target deconvolution method in *P. falciparum*. As expected, CETSA‐MS identified the target engagement of dihydrofolate reductase‐thymidylate synthase (PfDHFR‐TS) as the target for pyrimethamine whose target was known. CETSA‐MS identified purine nucleoside phosphorylase (PfPNP) as a probable target for quinine and a potential weak target for mefloquine.

Unbiased CETSA‐MS has recently been adapted to the field of antimalarial target deconvolution. The first example was using quinine and its derivative mefloquine (Figure 23).^223^ In this methodology, blood stage *P. falciparum* parasites and lysate samples were subjected to thermal melt or ITDR conditions; in all testing four separate experiment types. As CETSA had not been previously applied to *P. falciparum*, the melting properties of the proteome at trophozoite stage were characterized between 37°C and 73°C. *T*_m_ values could then be calculated for 80% (1821 proteins) of the trophozoite proteome, representing 65% of the overall blood‐stage proteome.^223^ Interestingly, proteins in infected RBCs had comparatively less thermal stability than their counterparts found in the lysate.^223^ Only 362 human erythrocyte proteins were characterized by this process, due to the disproportionate presence of hemoglobin which complicates the detection of peptides by MS.^224^ For the ITDR method, thermal challenge temperature was performed at 51°C to represent the average *T*_m_ for the proteome and 57°C for the fraction of the proteome that had greater thermal stability.^223^

To validate the method, ITDR and melt curve assays were performed in the presence of pyrimethamine (Figure 23), a known inhibitor of *P. falciparum* dihydrofolate reductase‐thymidylate synthase (PfDHFR‐TS).^225^ As expected, samples treated with pyrimethamine exhibited a temperature and dose‐dependent stabilization of PfDHFR‐TS.^223^ However, no such stabilization could be detected in treated infected RBCs.^223^ It was postulated that this could be the result of decreased affinity in a cellular context or due to the presence of a competing ligand such as folate. Validation of the infected RBC method was performed with the broad‐spectrum cysteine protease inhibitor, E64d.^226^, ^227^, ^228^ In this study, it was found that E64d stabilized four proteins, three of which were cysteine proteases (falcipain 2A, falcipain 3, and dipeptidyl aminopeptidase), while one was unexpectedly not a cysteine protease, the DSK2 protein homolog (PF3D7_1113400).^223^ The lack of thermal stabilization in cell lysate might also represent the necessity of the cellular environment for target engagement. This can include cellular drug activation, the availability of important cofactors, or the accumulation of the drug in a specific cellular compartment.^223^ Therefore, it is recommended to perform experiments with both lysate and whole cells to give greater confidence in the data.

ITDR was performed on cell lysate treated with quinine and MFQ (Figure 23) and purine nucleoside phosphorylase (PfPNP) was the only protein that showed a significant dose‐dependent stabilization.^223^ Ribosomal subunits and translation initiation factor 2 were also detected on treatment with MFQ, which is consistent with previous reports of its interaction with the ribosomal complex.^229^ In infected RBCs, PfPNP was similarly stabilized by quinine, but interestingly not MFQ.^223^ Instead, whole‐cell ITDR experiments with MFQ identified pyruvate kinase II (PfPyKII), although this may represent an increase in its abundance when cells are treated above 37°C and represent a downstream effect of drug binding or a stress response.^223^ Hsp70 and a GrpE protein homolog Mge1, two mitochondrial proteins, were also shown to be stabilized by MFQ but only at the highest dose.^223^ It was postulated that this may again be a result of an indirect effect on the mitochondrial membrane via reactive oxygen species formed by MFQ.^223^ Target engagement of quinine to PfPNP was confirmed by CETSA‐WB where dose‐dependent stabilization was again seen.^223^ Indeed, in vitro binding experiments by surface plasmon resonance (SPR) confirmed a *K*_d_ of 20 nM and 40 µM for quinine and MFQ, respectively.^223^ The enzymatic conversion of inosine to hypoxanthine by PfPNP was also found to be inhibited by quinine (*K*_i_ 138 nM) and mefloquine (*K*_i_ 5.9 µM).^223^ Overall, this data demonstrates PfPNP binds to quinine, but further investigation into the significance of PNP as the mechanism of action is required.

##### Plasmepsin IX and X inhibitors

**Figure 24:**
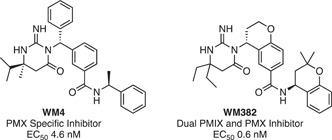
Structures of plasmepsin inhibitors and their specific targets. CETSA‐WB was used to confirm WM382 targets plasmepsin IX and X.

Alongside the chemical probe described earlier, Favuzza et al. demonstrated target engagement of their plasmepsin protease targeting compounds using CETSA‐WB.^159^ Resistance selection to the initial hit compound **WM4** (Figure 24) indicated that PMX was the target. However, the potent tool compound **WM382** appeared to have only a low level of cross‐resistance, indicating that it may have an additional target.^159^ It was hypothesized this additional target could be the closely related aspartyl protease, PMIX. As no recombinant PMIX was available at the time, CETSA‐WB was implemented to biochemically validate compound binding with HA‐tagged PMIX and PMX parasites. CETSA‐WB performed schizont purified parasite lysate successfully demonstrated that **WM382** indeed stabilized both PMIX and X, while the initial hit compound **WM4** stabilizes only PMX (Figure 24).^159^ The PMV inhibitor **W601**, used as a control did not induce the stabilization of either PMIX or PMX.^230^

### 4.2 Drug‐affinity responsive target stability

Drug‐affinity responsive target stability (DARTS) is a relatively new chemo‐proteomic technique used in target identification and validation. Similar to CETSA, the advantage of this technique is that it does not require modification of the compound of interest.^231^ DARTS is reliant on the decreased protease susceptibility generally observed upon binding of a ligand to its protein target.^232^ Therefore, the addition of proteases to drug‐treated lysate enriches target proteins in the mixture. DARTS is applicable to any cell type and has a relatively simple workflow.^233^ In DARTS, varying amounts of protease are used to determine a proteolysis curve, which is related to binding affinity.^234^ Enriched proteins are then detected either by western blot or mass spectrometry.^231^ Although DARTS is a robust method to demonstrate target engagement, the method has many of the same limitations as CETSA‐MS. One disadvantage is the binding affinity (*K*_d_) of the compound of interest may limit the effectiveness of the method, although DARTS has been successfully applied across a range of inhibitory concentrations.^234^ Another, is that protein targets involved with complexes or metabolic processes may result in the stabilization of proteins not bound by the compound of interest.^234^ A particular limitation of DARTS is that some proteins can be innately resistant to protease degradation.^235^

#### 4.2.1 DARTS examples

##### Torin 2

DARTS was used to complement the aforementioned AfBPP study to identify target proteins of the human mTOR inhibitor Torin 2 in gametocytes.^140^ A western blot from the DARTS experiment identified several protein bands that were stabilized in the presence of Torin 2 but not by the inactive Torin 1 control. The western blot bands were subsequently analyzed by mass spectrometry and revealed three proteins also found in the AfBPP study, phosphoribosyl pyrophosphate synthetase, aspartate transcarbamoylase (ATC), and a putative transporter (PF3D7_0914700).^140^ As mentioned, ATC was the only validated protein from these putative targets.^141^

### 4.3 Stability of proteins from rates of oxidation

An additional stability‐based technique developed for the purposes of target identification and validation is stability of proteins from rates of oxidation (SPROX). SPROX leverages the fact that ligand‐protein complexes usually reduce the rate of methionine oxidation compared to nonligated proteins.^236^ SPROX is considered more limited than CETSA and DARTS, as only proteins that contain multiple methionine residues may be targeted by this method, and the oxidation of methionines is not always mitigated by ligand binding.^237^ Additionally, nontarget proteins are not selectively degraded during the method, just chemically altered.^237^ Therefore, a negative enrichment of the target is not achieved and results may be more difficult to interpret. However, SPROX does possess a distinct advantage in proteins that act within multimeric complexes. Protein complexes are known to co‐aggregate in thermal profiling with similar melt curves, meaning that CETSA is unlikely to distinguish target engagement between these individual members.^238^

#### 4.3.1 SPROX examples

##### Clemastine

SPROX‐MS has been used alongside CETSA‐MS to determine the target of antihistamine clemastine.^239^ Clemastine was discovered to have antimalarial activity against both liver stage *P. berghei* and erythrocytic stage *P. falciparum*.^240^ However, *Plasmodium* does not encode any proteins with homology to the human target of clemastine, the histamine H_1_ receptor. Therefore, stability‐based techniques CETSA and SPROX were employed to determine the molecular target within the erythrocytic *P. falciparum* parasite.^239^ CETSA was able to identify a destabilization in the PfTCP‐1 ring complex (TRiC) in the presence of clemastine. The TRiC is an eight membered heterologous chaperone complex required for *de novo* cytoskeletal protein folding of actin and tubulin.^241^, ^242^ However, CETSA was unable to distinguish between the complex members with all eight being destabilized, perhaps due to the aforementioned co‐aggregation effect.^239^ With SPROX, compound‐dependent stabilization was observed in just one member of this complex, the delta subunit.^239^ This was confirmed biochemically, with the *K*_d_ to the delta subunit correlating well with the EC_50_ of the compounds in the parasites.^239^ The effect of this inhibition was also confirmed phenotypically with tubulin misfolding observed, leading to the disorientation of mitotic spindles.^239^

## 5 USE OF QUANTITATIVE PROTEOMICS

Proteomics‐based chemical biology techniques such as affinity pulldown and CETSA suffer from many practical issues with low‐affinity drugs and low‐abundance proteins.^98^ When these techniques are coupled with quantitative proteomics, the identification of putative targets is significantly more robust.^94^ Quantitative proteomics relies on differential heavy isotope labeling between peptide samples, which are then pooled and can be distinguished by mass spectrometry for relative quantification. Isotopically labeled peptide standards of known quantities may also be added to allow for absolute quantification. For example, heavy isotope labeling of either the control or active probes during affinity pulldown can help to establish specific protein interactions from nonspecific protein interactions the relative quantity of robust targets will be enriched only by the active probe. Enhanced detection of nonspecific binding can be important for low‐affinity binders as it reduces the reliance on excessively stringent washing techniques.^94^ For CETSA, the ability to perform relative quantification on a number of samples along a temperature or concentration gradient is central to demonstrating target engagement. Together with the ability to quantify relative peptide abundance, pooling samples together in one run serves to reduce instrument running time and variation between sample runs.^243^

### 5.1 Isotope‐coded affinity tagging

**Figure 25:**
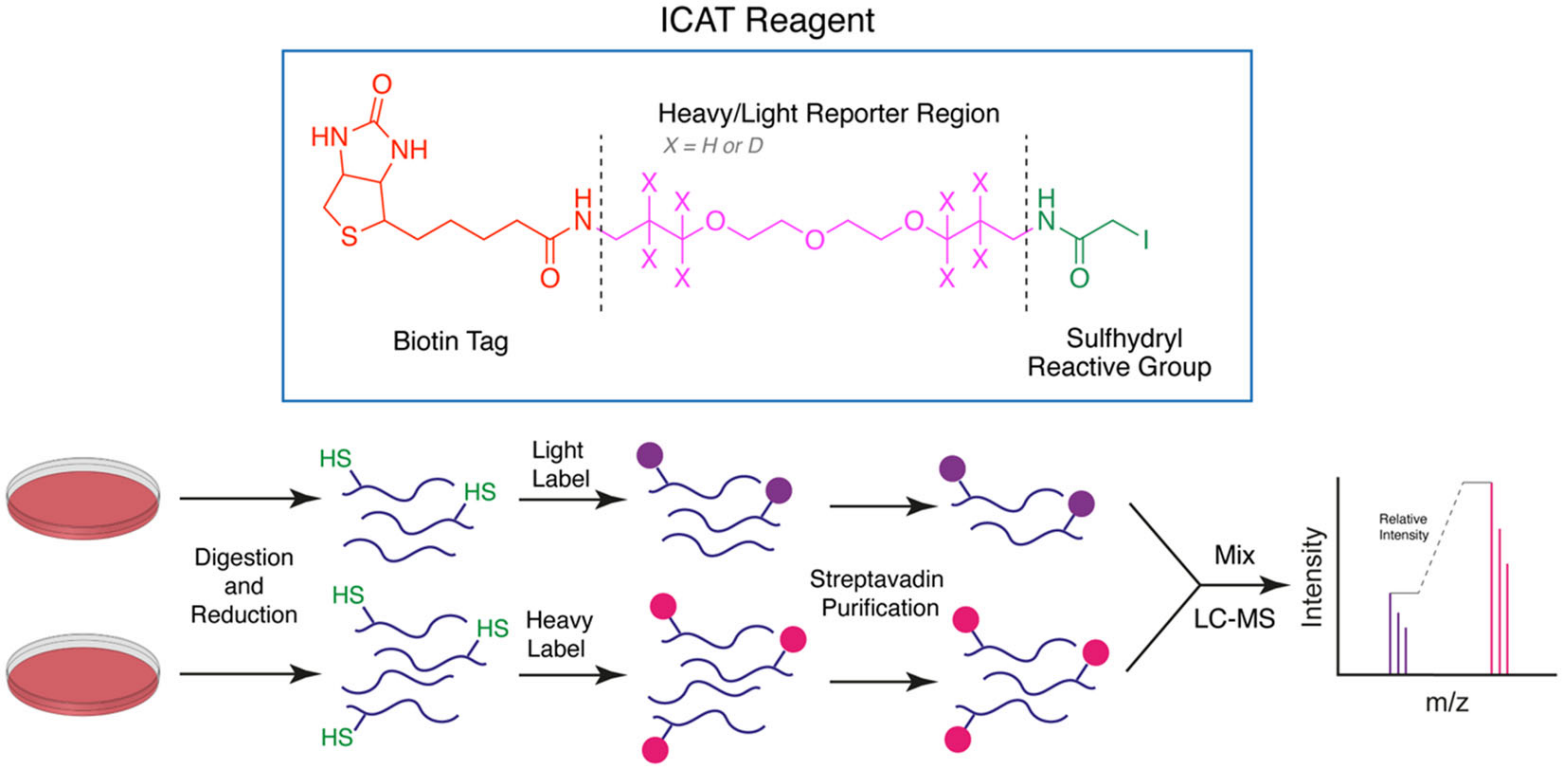
ICAT reagent and experiment workflow. The ICAT reagent contains a biotin tag for purification of labeled peptides, a heavy/light labeled PEG reporter region for mass spectral identification, as well as a sulfhydryl (cysteine) reactive group. ICAT is capable of differentially labeling two different samples which are first digested into tryptic peptides and reduced to expose sulfhydryl groups. The peptides are labeled with reagent and purified via streptavidin binding. The samples are then mixed and analyzed by mass spectrometry, where the relative intensity of the samples can be measured using differences in their mass. [Color figure can be viewed at wileyonlinelibrary.com]

The first example of these methods is isotope‐coded affinity tagging (ICAT). Stable isotopes do not alter the steric or physicochemical properties of a protein, but differences in mass are readily distinguishable through mass spec.^244^ Traditionally, ICAT uses isotopically labeled tags with a specific cysteine binding moiety and a biotin tag for purification (Figure 25).^245^ Proteins are then labeled with an isotopically heavy or light tag, digested, mixed, and analyzed mass spectrally (Figure 25).^246^ Proteins that are equally enriched in both heavy and light‐labeled samples are nonspecific binders, where specific enrichment in the active chemical probe sample is considered to correspond to the putative target.^245^ This technique is heavily reliant on the proportion of cysteine‐containing proteins within the proteome of interest. Cysteine residues represent just 1.7 mol% of the *P. falciparum* proteome and are present in only 17.5% of predicted tryptic peptides.^247^, ^248^ Therefore, many proteins may not be effectively captured by this technique. Additionally, incomplete labeling may occur, further reducing this further.^249^

### 5.2 Isobaric labeling methods

**Figure 26:**
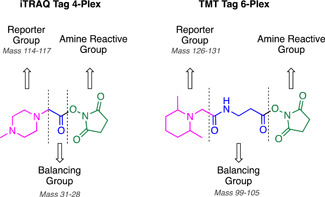
iTRAQ and TMT are methods of isobaric labeling for quantitative proteomic measurements. [Color figure can be viewed at wileyonlinelibrary.com]

Isobaric labeling is another quantification method that uses chemical labels to modify amino acid side chains of peptide samples. The labels are identical in mass that, upon fragmentation, yield reporter tags with differential heavy ^13^C and ^15^N isotope labeling and therefore mass. The general structure of these labels is a reporter group, a mass balancing linker grouping and an amine reactive group, NHS (Figure 26). iTRAQ (isobaric tagging for relative and absolute quantification) and TMT (tandem mass tagging) are examples of isobaric labeling techniques and follow essentially the same principle with minor differences in their label structure.^250^, ^251^, ^252^ iTRAQ is available to simultaneously quantify 4 or 8 samples concurrently,^253^ whereas TMT can quantify a larger range of samples with 2, 6, 8, or 10 different labels.^254^ Both of these methods can be adapted for absolute protein quantification by the addition of synthetic isobaric peptide standards.^251^, ^255^ Both the N‐terminus and lysine residues are labeled by isobaric labeling methods; therefore, they are widely applicable to a majority of peptides and proteins in all cell types.^243^ Multiplexing other steps into the protocol, such as 2D liquid chromatography and TiO_2_‐mediated phosphopeptide enrichment, may be useful in quantifying post‐translationally modified proteins.^256^

Isobaric labeling methods have been applied in combination with the aforementioned chemical biology techniques for the purposes of antimalarial target identification. TMT labeling has been used by Chibale et al. in the target identification of MMV048 following pulldown with a Sepharose‐linked probe and by Nordlund et al. 2019 for CETSA‐MS to identify targets of quinine, mefloquine, and pyrimethamine.^137^, ^223^ Additionally, 4‐plex iTRAQ has been used alone as a method of target identification by chemoproteomics to monitor the relative expression levels of *P. falciparum* proteins following treatment with Doxycycline.^257^ Treatment resulted in the differential expression of 40 distinct proteins, many localized to the mitochondria and apicoplast organelles with functions of protein synthesis and processing.

### 5.3 Stable isotope labeling with amino acids in culture

The gold standard method of quantitative proteomics is called stable isotope labeling with amino acids in culture (SILAC). This method uses heavy isotope labeling of essential amino acids, supplied in culture to cells of interest to distinguish between conditions.^258^ Relative quantification is achieved by supplementing one sample with natural amino acids, while the other receives amino acids with heavy ^13^C or ^15^N isotope labels. These amino acids are incorporated into newly synthesized proteins, therefore unlike other methods of quantitative proteomics, isotopic labeling is achieved before the experiment. SILAC provides gold standard relative quantification for several reasons. The workflow is compatible with the majority of cell types and beyond routine cell culture, requires no specialized treatment.^259^ Labeling occurs in a complete manner and sample mixing occurs at the cellular stage, meaning that MS preparation steps such as purification and protein digestion are done on a uniform sample of pre‐labeled proteins.^260^ Other labeling strategies involve the mixing of samples following the preparation of the peptides for MS, meaning that sample loss can occur in these handling steps, affecting the relative quantities of samples.^260^

SILAC was developed for *P. falciparum* in 2004 by Nirmalan et al. to overcome challenges inherent to *Plasmodium*.^247^ Amino acids are sourced via several avenues: digestion of erythrocyte hemoglobin, de novo synthesis as well as import from the host erythrocyte. Isoleucine is the only amino acid not present in human hemoglobin; therefore, it is obtained entirely from exogenous sources.^261^ This makes it uniquely suited to quantitative heavy isotope labeling in *Plasmodium.* Unlike cysteine, isoleucine is also highly abundant, representing 9.2 mole% of the proteome and is present in 60% of tryptically digested peptides.^247^ A difference of 7 Da is observed between ^13^C_6_–^15^N_1_ and ^12^C_6_–^14^N_1_ isotopes which provides excellent spectral separation.^262^ The authors use this newly developed method to identify targets of pyrimethamine and tetracycline through differential proteome analysis of *P. falciparum* parasites.^247^ The method has since been applied to the proteome analysis of chloroquine and artemisinin treatment,^262^ but has not been used in combination with chemical biology‐based target identification. There are perhaps a few reasons for this. Although lysine and arginine isotopologues have been commercially developed for SILAC multiplexing of up to four samples,^263^ no such technology is available with isoleucine for the above method in *P. falciparum*. This greatly limits experimental design and precludes use with CETSA altogether. The expense of SILAC's isotopic reagents also hinders its widespread use in the laboratory.^103^

### 5.4 Label‐free methods

The most widely applied methods of quantitative proteomics in malaria drug discovery are label‐free methods. While label‐based methods are considered to be more accurate, expensive reagents, a limited number of samples, and limits in sample applicability make label‐free methods particularly attractive.^264^ Label‐free methods rely on liquid chromatography‐tandem mass spectrometry (LC‐MS/MS) and are based on two methods of detecting peptide abundance: ion intensity and spectral number.^265^

Spectral counting uses the number of MS/MS events corresponding to a single peptide to measure protein enrichment.^266^ A higher abundance of protein will result in a greater number of tandem MS spectra generated.^267^ More accurate quantification is enabled by correcting for overall protein length (NSAF, dNASF, and SI_N_).^268^, ^269^, ^270^ or by the theoretical number of tryptic peptides (emPAI and APEX).^271^ An advantage of spectral counting is its simplified workflow. Unlike in ion intensity measurement, computational pre‐processing of data is not required and therefore can be employed immediately.^272^ However, the primary issue with spectral counting is the presence of nonunique peptides or those shared by multiple proteins. It has been estimated that around 50% of tryptic peptides identified in databases are nonunique and some proteins are entirely composed of nonunique tryptic peptides.^272^ This has previously been accounted for by excluding them from the analysis altogether, distributing them to proteins based on another property such as the abundance of other corresponding unique peptides, or by ignoring that they are shared and counting them multiple times.^264^ The latter method is now considered inappropriate as many proteins will not be accurately captured.^273^ emPAI has been extensively used in quantitative proteomics for antimalarial research^154^, ^164^, ^165^, ^174^, ^183^, ^199^ as it is incorporated into the protein identification search engine Mascot.^274^

The ion intensity method relies on measuring the area of an MS1 peak (AUC) at a given mass‐to‐charge ratio (*m/z*). A linear correlation exists between this area and peptide concentration (*r* ^2^ = 0.991–0.9978), allowing for direct comparison between identical peptides for accurate determination of their relative quantity.^275^, ^276^ Approximate quantification of peptides is enabled through several normalization methods. Like spectral counting methods, normalization can be achieved by accounting for the number of theoretical peptides (iBAQ and riBAQ),^277^, ^278^ the molecular mass of the protein (TPA),^279^ or taking the intensity of the top three most intense peptides (TOP3).^280^ MS/MS spectra must also be subsequently obtained to confirm the identity of each peptide. In complex peptide mixtures, it is necessary to carefully optimize the statistical and computational parameters of quantification.^281^ In particular, technical variations in peptide retention time, co‐elution, and background noise are concerns of this method.^265^ High‐resolution mass spectrometers and computational methods aid in aligning data between runs.^267^ Programs MaxQuant and Progenesis QI have been used in antimalarial target ID to determine relative quantification through AUC.^173^, ^185^, ^211^ This is considered a main disadvantage of AUC as it adds significant complexity to experimental optimization and quantification can be variable depending on which algorithm is used.^272^ However, overall AUC is considered more accurate than spectral counting due to the higher stochasticity of [MS/MS] methods.

## 6 FUTURE DIRECTIONS

Chemoproteomic‐based target deconvolution is rapidly evolving with older technologies being incrementally improved and new technologies being developed. To optimize the application of chemical probes, it would be beneficial to develop a plasmodial database similar to the human CRAPome which acts as a repository for common contaminant proteins in affinity purification–mass spectrometry.^156^ Meanwhile, other methods remain to be applied to full antimalarial target deconvolution, such as PROTACs.

### 6.1 *Plasmodium* CRAPome

As has been previously highlighted, nonspecific binding to the support resin is a prominent issue for affinity‐based protein purification techniques. While inactive control probes can effectively account for this issue, the availability of these controls can at times be limited. Negative controls taken at an individual level may also be sensitive to small variations in sample preparation and fail to give a complete picture of nonspecific binding. Fortunately, nonspecific binding is largely independent of the bait molecule and more likely due to the chosen resin. With this in mind, the CRAPome or the Contaminant Repository for Affinity Purification was created. This database collates and annotates published MS proteomic data derived from negative control probes which could be used to score query MS data.^156^ This database currently exists only for the human, mouse, drosophila, yeast, and *E. coli* proteomes with a range of resin types.^282^ Given that affinity‐based purification is extensively used as a target deconvolution method in *Plasmodium*, we feel that a similar database would be beneficial to the identification of high‐quality targets.

### 6.2 Proteolysis‐targeting chimeras

**Figure 27:**
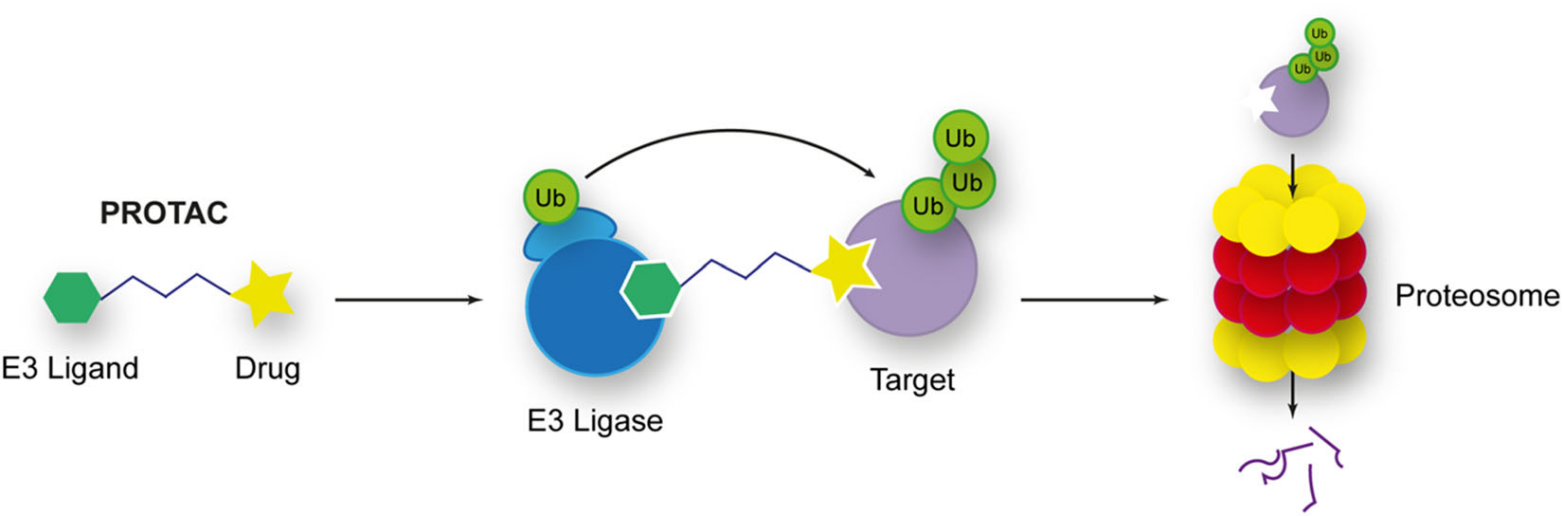
Mechanism of action of proteolysis‐targeting chimeras (PROTACs). PROTACs are heterodimeric bifunctional molecules that link an E3 ligand to a drug molecule. By doing this, they bring into proximity a target with complexes that polyubiquitylate it and target it for degradation by the proteasome. Coupled with mass spectrometry, these molecules can detect the targets of drug molecules identified by phenotypic screening. [Color figure can be viewed at wileyonlinelibrary.com]

Proteolysis‐targeting chimeras (PROTACs) are bifunctional molecules that artificially enhance the clearance of a protein by recruiting cellular machinery that facilitates its degradation.^283^ They can be considered a kind of modified chemical probe, containing a drug pharmacophore conjugated via a linker to an E3 ligase binding moiety (Figure 27). They bring into proximity the protein target of a compound with machinery that can ubiquitylate it and thereby destine it for proteasomal degradation. Combined with mass spectrometry PROTACs have great potential for the identification and validation of drug targets, acting as a form of chemical knockdown which is fast, dose‐dependent, and reversible.^284^ They have been used previously to identify the targets of phenotypically discovered anticancer compounds in human cells,^285^, ^286^ but not yet in *Plasmodium*. *Plasmodium* possesses homologs of the eukaryotic and prokaryotic proteosomes as well as the cyanobacterial Clp protease.^287^ There are several E3 ligase proteins present in the *P. falciparum* proteome, with RING (really interesting new gene) finger E3's being the most abundant.^288^ It has been shown that *Plasmodia* rely heavily on protein degradation for development and stress response in all lifecycle stages.^289^ While a functional ubiquitin proteasomal degradation system almost certainly exists in *Plasmodium*, future work in this space will require multi‐omic characterization of E3 ligases, the design of suitable E3 ligands, and by extension, the PROTAC molecules themselves.^290^ The high molecular weight, lipophilicity, rotatable bonds, and polar surface area necessitated by heterodimeric bifunctional compounds can be a barrier to cell permeability and therefore their utility in cell‐based assays.^291^ Therefore, there is some time before the widespread use of this technology in *Plasmodium* parasites.

## 7 CONCLUSIONS

With resistance arising to currently available antimalarials and many of those undergoing clinical development, the need for novel therapeutics continues. High‐throughput screening continues to be a prominent method by which these chemical entities are discovered. This necessitates robust and informative techniques for target identification to determine the drug mechanism of action. Today, target identification necessarily pulls techniques from many disciplines to deconvolute complex protein mixtures. Chemical biology represents an emerging field in target identification and engagement that is complementary to a range of others, such as drug resistance and metabolomics. It is an incredibly direct technique for target identification and engagement, providing an explicit link between the chemical entity and the biological effect. For these reasons, it has been extensively applied to the field of antimalarial drug discovery.

Chemical probes have proven to be diversly applicable across chemotypes and mechanisms of action. Therefore, it is unsurprising that antimalarial target identification has heavily relied on this technique. An evolution in chemical structure from the traditional Sepharose‐conjugated probes has enabled this expansion in functionality. Biocompatible functional tags such as biotin or alkyne/azide click chemistry handles have enabled their use in live cells, rather than using cell lysate with a resin‐tethered probe. Similarly, fluorescently tagged probes allow valuable information on compartmental localization in live cells to be gained. Photo‐crosslinking probes enhance the sensitivity of methods in Sections 3.2 and 3.4 and have been utilized in complex, multifunctional structures (Table 1) Overall, chemical probes are an information‐rich target identification method that must be employed with careful controls and verified through other distinct approaches.

**Table 1:** Summary of chemo‐proteomic methods used in target identification and engagement.

Method		Method strengths	Method weaknesses	Exemplar and target	Refs.
AfBPP	− Compound competition can improve target deconvolution − Coupled with bioorthogonal conjugation, it is applicable to whole parasites		− Requires SAR to label − Requires highly potent and selective compound to pulldown target successfully − Label can reduce affinity of probe − Prone to pulling down highly abundant promiscuous proteins	WM382/PfPMX	^159^
ABPP	− Reactive warhead usually has high potency for target(s) − Compound competition can improve target deconvolution − Coupled with bioorthogonal conjugation is applicable to whole parasites		− Requires SAR to label − Usually requires bead digestion to release covalent proteins − Non‐selective reactivity can be prone to pulling down many proteins.	Salinipostin A/ten Pf α/β serine hydrolases	^178^
Photo‐affinity BPP	− Photoreactive group enhances ability to covalently pulldown targets catalytic reactive residues. − Compound competition can improve target deconvolution − Coupled with bioorthogonal conjugation is applicable to whole parasites		− Requires SAR to label − Non‐specific photo labeling with highly abundant proteins − Requires suitable irradiation apparatus	(Z‐LL)_2_/*Pf*SPP	^193^
CETSA‐MS	− Label‐free − Applicable with whole or lysed parasites		− Typically requires high compound concentration − Prone to detecting physiologically nonrelevant proteins − Limitations with detecting proteins in multimeric complexes	Quinine/PfPNP	^223^
DARTS	− Label‐free − Protease addition enriches targets		− Typically requires high compound concentration − Not applicable for detecting protease‐resistant proteins	Torin 2/PfATC	^140^
SPROX	− Label‐free − Can detect proteins in multimeric complexes		− Requires proteins with multiple methionines − Oxidation of methionines can be heterogeneous	Clemastine/PfTRiC	^239^

Stability‐based techniques such as CETSA, DARTS, and SPROX have been used with success in antimalarial target identification. These are relatively new methods to measure target engagement that does not require the modification of the drug of interest. For CETSA, the ability to assess target engagement in the context of both cellular lysates and in live cells provides a biologically relevant result. However, their lack of applicability to all targets remains an issue. Membrane‐bound targets and those involved with complex multi‐protein oligomers or pathways can provide misleading results (Table 1).

The advent of chemical biology target deconvolution owes its roots to advancements in proteomics, in particular mass spectrometry modified to allow quantitative assessment. Isotopic labelings, including ICAT, iTRAQ, TMT, and SILAC as well as label‐free methods, allow for relative or absolute quantification of protein levels across different samples. As chemical biology often involves positive or negative enrichment at the proteomic level, these techniques provide more accurate and sensitive results than traditional 2‐DE gel proteomics.

Finally, there are several other chemical biology techniques that have not yet been used for the purposes of antimalarial target deconvolution such as PROTACs. These methods, including PROTACs, could represent a new area of development for antimalarial research in terms of both therapeutics and target deconvolution.

## Data Availability

Data sharing is not applicable to this article as no new data were created or analyzed.
